# A specifically designed multi-biotic reduces uremic toxin generation and improves kidney function

**DOI:** 10.1080/19490976.2025.2531202

**Published:** 2025-07-12

**Authors:** Alice Beau, Jane Natividad, Berengère Benoit, Philippe Delerive, Stéphane Duboux, Yuan Feng, Marie Jammes, Cecile Barnel, Giuseppina Sequino, Claudie Pinteur, Griet Glorieux, Denis Fouque, Hubert Vidal, Laetitia Koppe

**Affiliations:** a CarMeN Laboratory, INSERM, INRAE, Claude Bernard Lyon 1 Université, Pierre-Bénite, France; b Nestle Health Science, Lausanne, Switzerland; c Nestlé Research, Lausanne, Switzerland; d Department of Nephrology and Nutrition, Hospices Civils de Lyon, Centre Hospitalier Lyon-Sud, Pierre-Bénite, France; e Department of Agricultural Sciences, University of Naples Federico II, Portici, Italy; f Nephrology Unit, Department of Internal Medicine and Pediatrics, Ghent University Hospital, Gent, Belgium; g Lyon GEM Microbiota Study Group, Lyon, France

**Keywords:** Multi-biotic, chronic kidney disease, uremic toxins, gut microbiota

## Abstract

Chronic kidney disease (CKD) is characterized by accumulation of uremic toxins (UTs), such as p-cresyl sulfate and indoxyl sulfate, generated through the transformation of tyrosine and tryptophan by the gut microbiota. Using an *ex vivo* Simulator of the Human Intestinal Microbial Ecosystem (SHIME) colonized with fecal samples from eight CKD patients or nine healthy volunteers, a higher bacterial generation of p-cresol and indoles post-amino acid enrichment, as well lower basal butyrate levels, in the feces of CKD patients were found. Through *in silico* data mining, we selected a probiotic strain lacking the capacity to produce UT, i.e. without genes for tryptophanase, tyrosinase and urease. *In vitro*, we confirmed the potential of cellobiose as a prebiotic supporting the growth of this strain. We further designed a novel specific multi-biotic for CKD (SynCKD) [containing a probiotic *Lactobacillus johnsonii NCC533*, a prebiotic (1% cellobiose), and a postbiotic (1% short and medium chain triglycerides C4-C8, a source of butyrate)]. SynCKD effectively curtailed UT precursor generation *ex vivo*. The *in vivo* efficacy of SynCKD (and the synergic effect) was established in two uremic rodent models, demonstrating lower plasma levels of UTs and enhancing kidney function after 6–8 weeks of treatment. These effects were linked to better gut microbial ecology. Metagenomic analysis revealed reduced microbial genes for tryptophan/tyrosine degradation. This study lays the foundation for SynCKD as a potential therapy to mitigate CKD progression.

## GRAPHICAL ABSTRACT

**Figure:**
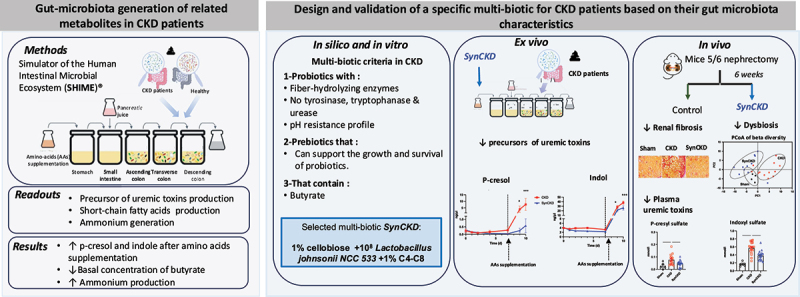

## Introduction

Chronic kidney disease (CKD) is a prevalent global health issue, affecting nearly 10% of the worldwide population.^1^ Given the lack of specific symptoms in the early stages of the disease, timely detection and treatment of CKD pose significant challenges. CKD progression and adverse outcomes in CKD patients are, at least in part, the consequence of the high levels of deleterious bioactive compounds called uremic toxins (UTs).^2^

A number of therapeutic strategies targeting UTs have been proposed, including reducing substrate levels (such as protein restriction), decreasing absorption (using oral adsorbents like AST 120), increasing clearance through renal replacement therapies (e.g., enhanced dialysis, absorbent membranes, kidney transplantation), and modulating cellular pathways (such as organic anion transporters and antioxidants). However, most of these treatments present significant limitations, such as side effects, high costs, and limited availability for patients with non-terminal CKD, and they remain largely confined to experimental settings. Therefore, new therapeutic approaches need to be explored and developed. Given that the majority of UTs were found to originate from the gut microbiota, the latter appears as a new and attractive therapeutic target.

The accumulation and high level of UTs in the bloodstream of CKD patients results from impaired kidney function (reduced clearance), and increased *de novo* generation due to CKD-associated dysbiosis.^3^ Furthermore, although it is not clearly demonstrated, increased gut permeability leading to a higher absorption of UTs, as observed in *in vitro* ^4^ and *in vivo* ^5,6^ models, may also play a role.^7^

Among the UTs that are the most frequently described to be associated with poor outcomes, especially for the cardiovascular system, are p-cresyl sulfate (PCS), p-cresyl glucuronide (PCG), indoxyl sulfate (IS), and indole-3-acetic acid (IAA). All of these arise from the microbial metabolism of aromatic amino acids (aromatic AAs; tyrosine, phenylalanine, and tryptophan).^8^ CKD patients often also have elevated levels of trimethylamine N-oxide (TMAO), a toxic gut-derived metabolite and a product of the gut microbial metabolism of dietary choline and carnitine.^9^ Furthermore, CKD patients demonstrate a noticeable reduction in the abundance of bacteria responsible for generating short-chain fatty acids (SCFAs), specifically butyrate, which is associated with a low fecal concentration of butyrate.^5,10^ These are instrumental in maintaining intestinal mucosal integrity, regulating metabolism, controlling energy expenditure, and modulating the immune system.^11^ Moreover, they potentially slow-down kidney function decline.^5,6,12^ Hence, efforts to reduce plasmatic UT levels as well as prevent their accumulation and absorption, while preserving the balance between beneficial metabolites such as SCFAs and toxic gut-derived metabolites, are of utmost importance.

Discrepancies exist regarding the contribution of gut microbiota in explaining the high plasmatic UT levels in CKD patients.^13,14^ For example, Gryp et al.^15^ did not observe an increase in UT precursor levels in fecal samples from CKD patients compared to healthy volunteers (HV), concluding that the elevation in plasma UT concentrations was largely attributed to a decrease in kidney function. Also, similarly, the metabolic deregulation of hydrogen sulfide was not associated with modifications in gut microbiota composition in CKD patients.^16^ Conversely, Wang et al.^17^ found, in CKD patients, a correlation between fecal UTs precursors and plasmatic UTs concentration but also the abundance of genes encoding UT-synthesizing enzymes in the gut microbiota. These differences may be explained by the methodological approaches used.^15,17^

Several proof-of-concept studies have suggested the potential benefits of probiotic and prebiotic consumption in modulating the gut microbiota of CKD patients.^18–20^ However, a recent meta-analysis found limited evidence supporting biotic supplementation in CKD management, likely due to the empirical selection of both prebiotic and probiotic strains, synbiotics (a combination of pro, and prebiotics) or multi-biotics (a combination of pro, pre, and postbiotics.^21^ For instance, probiotics containing urease enzymes may exacerbate the generation of ammonia from urea, while the uremic environment in the gut may curtail the survival and health benefits of probiotic strains.^18^ In addition, the compatibility between probiotics and prebiotics is a crucial consideration when formulating synbiotics, especially because the interactions between the gut microbiota and a probiotic strain can vary according to the prebiotic used.

Taken together, the specific contribution of the microbiota to the high plasmatic levels of UTs remains unclear to date. It is important to clarify this point to determine whether the use of therapeutics that modulate the microbiota, such as multi-biotics, is worthwhile. Therefore, in the present study, the first aim was to explore the specific contribution of gut microbiota sourced from individuals with CKD to the generation of major gut microbiota-derived metabolites. This was achieved *ex-vivo* by using a gastrointestinal model colonized with fecal samples obtained from CKD patients or HV. To isolate the influence of diabetes and obesity on gut microbiota composition and function, we intentionally selected patients without diabetes and obesity. The second aim was to carefully select and design a specific multi-biotic (combination of pre-, pro-, and postbiotics) for CKD, using *in silico* and *in vitro* strategies, with the primary objective to reduce the UTs production such as IS and PCS by influence of gut microbiota composition and function. We tested its interactions with gut microbiota and the generation of metabolites in this *ex vivo* method. Finally, we determined the potential benefits of daily oral administration of the designed multi-biotic for 6 to 8 weeks on intestinal homeostasis, UTs, and the kidney function in rodent models of CKD.

## Methods

See the online supplementary methods for more details.

### In silico screening of industrially available probiotic strains

AA sequences of all predicted proteins from probiotic strains were retrieved from the RefSeq database, and the AA sequences of the enzymes for each reconciled pathway were extracted from UniProt.

### Growth profile of Lactobacillus johnsonii NCC 533 on cello-oligosaccharides

*L. johnsonii* NCC 533 was obtained from Nestlé, Switzerland. The growth profile of *L. johnsonii* NCC 533 with different carbohydrates (cellobiose, cellotriose, cellotetraose) was performed in a BioLector microbioreactor (m2p-labs, Baesweiler, Germany). Biomass was recorded over the incubation period.

### Collection of fecal samples from HV and CKD donors

Ten non-diabetic stage four to five CKD patients and ten HV were recruited (NCT04768309, CPP Sud EST II, N° 202–007). The selection criteria were: ability to provide fresh feces at a particular time, and, for CKD patients an estimated glomerular filtration rate (eGFR) <30 ml/min/1.73 m^2^, without diabetes, inflammatory disease, obesity (Body mass index (BMI) between 18–30 kg/m2), as well as no antibiotic, laxative, probiotic or prebiotic during the previous month), and, for HV, eGFR ≥ 90 ml/min/1.73 m^2^, no albuminuria, no treatment, and no history of kidney injury. The diet profile was quantified using the last 3-day recall. Fecal samples were transferred under anaerobic conditions and were placed inside a hermetic glass box and kept at 4°C. Within 24 h, fecal suspensions were prepared, mixed with an in-house optimized cryoprotectant, and stored at −80°C until use.^22^

### Treatment in the SHIME

**Figure 1.**
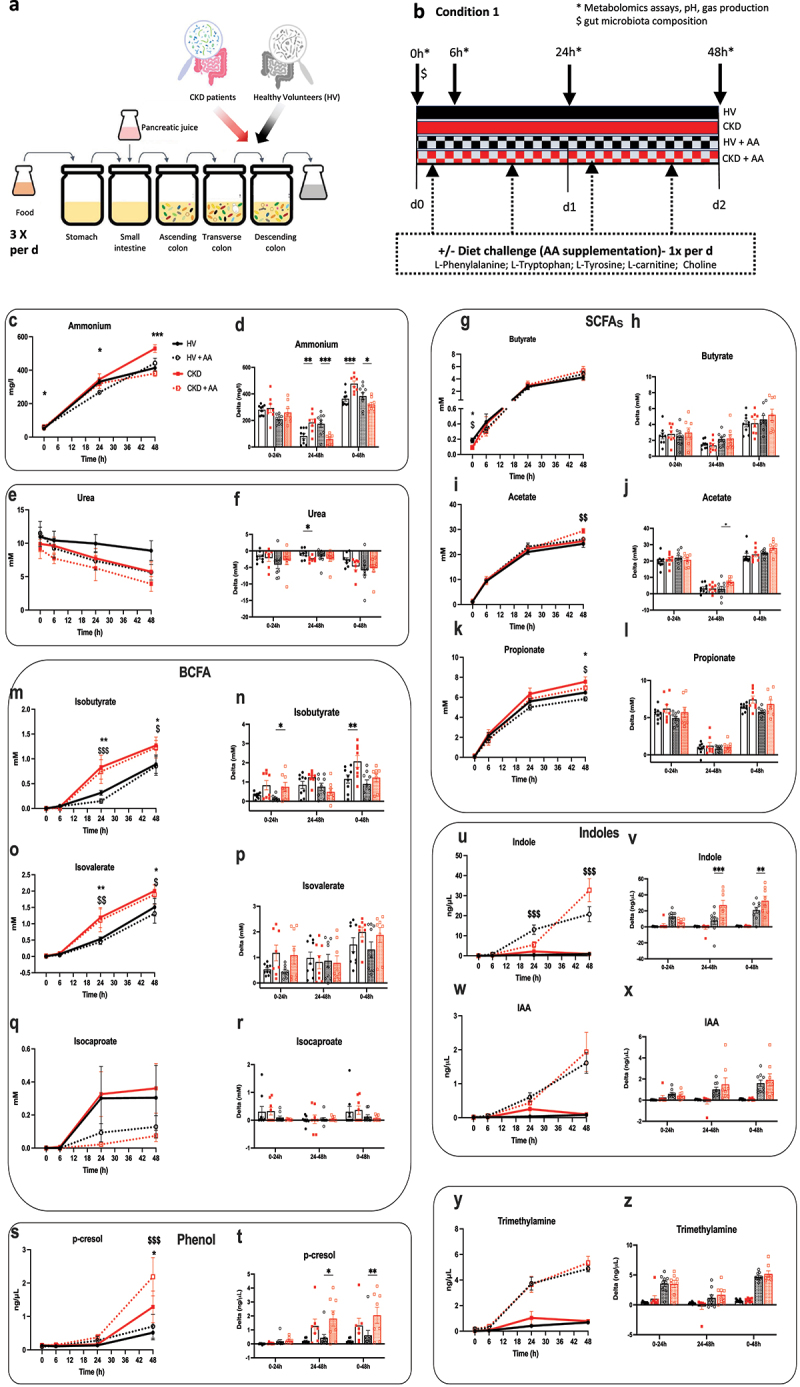
*Ex vivo* generation of gut-derived metabolites using the simulator of human intestinal microbial Ecosystem (SHIME) system, colonized by feces from chronic kidney disease (CKD) patients and healthy volunteers (HV) during a 48 h period with or without amino acid (AA) supplementation in the diet challenge.

The setup of the Simulator of the Human Intestinal Microbial Ecosystem (SHIME) (ProDigest, Ghent, Belgium) has been previously described.^23^ Briefly, it consists of a succession of five reactors simulating the different parts of the human gastrointestinal tract (Figure 1(a)). The first two reactors are of the fill-and-draw principle to simulate different steps in food uptake and digestion, with peristaltic pumps adding a defined amount of SHIME feed (140 ml 3x/day; 1 g l^−1^ arabinogalactan, 2 g l^−1^ pectin, 1 g l^−1^ xylan, 3 g l^−1^ potato starch, 0.4 g l^−1^ glucose, 3 g l^−1^yeast extract, 1 g l^−1^ pepton, 4 g l^−1^ mucin, 0.5 g l^−1^ cystein) and pancreatic and bile liquid (60 ml 3x/day), respectively, to the stomach and small intestine compartment and emptying the respective reactors after specified intervals. The last three compartments simulate the large intestine. These reactors are continuously stirred, they have a constant volume and pH control. The colon reactors were inoculated with a fecal sample. After one day of stabilization, two experiments were conducted. In the condition 1: HV were compared to CKD for 48 h. We have four groups: the control arm of each donor (CKD and HV) and the diet-challenging group of each donor supplemented additionally with an AA mix (CKD + AA and HV + AA). The AA mix was added during one of the feeding cycles (i.e., once a day; see Supplementary Table S1 for composition). The AA mix was selected to explore more relevant bacterial pathways involved in CKD mechanisms, such as p-cresyl sulfate/p-cresyl glucuronide from tyrosine and phenylaniline metabolism, indoxyl sulfate from tryptophan metabolism, and TMAO from choline and carnitine metabolism. In the condition 2: CKD were compared to CKD + multi-biotic for an 11-day period. Details of *ex-vivo* studies, including feces preparation, feeding of the SHIME reactor, and treatment with the multi-biotic are provided in the online supplementary materials.

(a) SHIME system was colonized with a cryostock containing an inoculum of each donor. (b) Chronogram of the experiment (condition 1). Kinetic and delta profiles during a 48 h period with and without a diet challenge of (c-d) ammonium (e-f) urea, (g-h) butyrate, (i-j) acetate, (k-l) propionate, (m-n) isobutyrate, (o-p) isovalerate, (q-r) isocaproate, (s-t) p-cresol (u-v) indole, (w-x) indole acetic acid (IAA), and (y-z) trimethylamine (*n* = 8–9). Data are mean ± standard error of the mean. *p < 0.05, **p < 0.01, and ***p < 0.001, respectively, for CKD *versus* HV. $ *p* < 0.05, $$ *p* < 0.01, and $$$ *p* < 0.001, respectively, for CKD + AA *versus* HV + AA. Two-way ANOVA with Bonferroni *post hoc* test was performed for kinetic and one-way ANOVA with Bonferroni *post hoc* test for AUC.

### Measurement of SCFAs, ammonium, and metabolites

SCFAs were measured via a GC-FID method.^24^ Ammonium was quantified via steam distillation.^25^ *In vitro* fluids and blood samples were analyzed using a targeted metabolomics approach based on Ultraperformance Liquid Chromatography – High-Resolution Mass Spectrometry (UHPLC-HRMS), as previously described.^26,27^

### Animals experiments

All animal studies were approved by the local ethics committee (#LS_2020_004 et 2020_005 and APAFIS#30603–2021012517248777 v3) as well as the Danish Animal Experimentation Council, and conformed with the ARRIVE guidelines. Two independent animal studies were performed to test the role of the multi-biotic in renal disease. Briefly, to induce CKD, the upper and lower poles of the right kidney were resected by electro-coagulation to induce. One week later, the left kidney was removed after ligation of the kidney blood vessels and the ureter. Special care was taken to avoid damage to the adrenals. Details of animal studies, including sample collection, quantitation of UTs, as well as histological and immunohistochemical analyses, are available in the online supplementary materials.

### Metagenomic sequencing

Total fecal DNA in the cecum of mice was extracted using standard methods.^28^ Libraries were prepared using the NEBnext Ultra II DNA Library Prep Kit for Illumina (New England Biolabs, Évry-Courcouronnes, France). The Illumina HiSeq X Ten platform (New England Biolabs) was then used for 2 × 150 bp paired-end whole-metagenome sequencing. The number of total bacterial cells were quantified using flow cytometry.^29^

### Statistical analysis

Statistical analyses were performed in the R environment (https://www.r-project.org.) and GraphPad Prism 10 (https://www.graphpad.com), and all methods are detailed in the online supplementary materials.

## Results

### The fecal microbiota of CKD patients generates more ammonium, branched-chain fatty acids (BCFA), indoles, and phenols in ex vivo incubations than HV

Among the ten CKD and ten HV included in the study, respectively, eight CKD patients and nine HV were analyzed. Although there was a significant difference in age, they were similar in terms of BMI, sex distribution, and food intake. As expected,^30^ CKD patients exhibited kidney dysfunction as well as elevated plasma levels of UTs (Table 1). We observed a significant variation in microbial diversity and taxonomic composition between CKD patients and HV (Figure S1). In the condition 1, the SHIME was colonized with fecal sample from each donor (Figure 1(a)) and the system was supplemented with aromatic AAs, choline, and carnitine, at concentrations representative of a normal protein diet as recommended for human health^31^ (Figure 1(b) and Supplementary Table S1).

**Table 1.** Study population characteristics.

	Healthy volunteers	CKD	*P* value
No. of patients	9	8	
**Characteristics**			
Age, years	37 ± 8	55.2 ± 3.4	< 0.001
Sex, F/M	2F/7 M	2F/6 M	0.89
BMI, kg/m^2^	23.6 ± 2.5	23.8 ± 1.0	0.53
**Treatment, n**			
Lipid-lowering therapy	0	5	
RAA system antagonists	0	5	
PAA agents	0	0	
Potassium chelator	0	3	
Proton pump inhibitor	0	0	
**Cause of nephropathy**			
Glomerulopathy, n	NA	5	
Urology (n)	NA	3	
**Kidney function**			
Creatinine (μmol/l)	85 ± 12	309 ± 17.8	< 0.001
eGFR ml/min/1.73 m2	101 ± 3.5	18 ± 1.1	< 0.001
Urea, mmol/L	4.8 ± 0.4	17.9 ± 1.2	< 0.001
Proteinuria, g/24 h	0 ± 0	1.41 ± 0.4	< 0.001
**Inflammatory parameters**			
CRP, mg/L	2.1 ± 1.1	5.1 ± 3.4	0.39
IL6, pg/ml	1.1 ± 0.1	13.4 ± 11.1	0.28
TNF alpha, pg/ml	7.4 ± 0.9	18.6 ± 0.7	< 0.001
**Food intake**			
Proteins, g/kg/day	0.95 ± 0.08	0.87 ± 0.07	0.45
Calories, kcal/kg/day	25.6 ± 2.3	22.5 ± 2.5	0.37
Fiber g/day	17.1 ± 1.8	14.1 ± 2.2	0.31
**Plasmatic uremic toxins**			
IS, μM	9.8 ± 1.9	34.0 ± 0.05	< 0.001
PCS, μM	15.3 ± 3.2	43.6 ± 7.1	< 0.001
PCG, μM	0.1 ± 0.1	0.7 ± 0.2	0.05
IAA, μM	3.4 ± 0.2	4.4 ± 0.4	0.09

Data are presented as mean ± standard error of the mean and categorical data are presented as n.

Abbreviations: BMI: Body Mass Index; CRP: C-Reactive Protein; eGFR: Estimated Glomerular Filtration Rate; IL: Interleukin; IS: Indoxyl Sulfate; IAA: Indole-3-Acetic Acid; NA: not applicable, PAA: Platelet Aggregation Agent; PCS: p-Cresyl Sulfate; PCG: p-Cresyl Glucuronide; RAA: Renin-Angiotensin-Aldosterone, TNF: Tumor Necrosis Factor Differences between groups were tested using Student t test or Chi-squared test as appropriate.

The fermentation capacity of the gut microbiota was assessed through gas generation, pH monitoring, and lactate measurement (Supplementary Figure S2a-f). Over the 48 h of incubation, among CKD patient samples without AA supplementation there was a significant decrease in pH (Supplementary Figure S2 d), related to elevated ammonium generation (Figure 1(c,d)) and decreased urea concentration (Figure 1(e-f)).

Basal fecal levels of butyrate were significantly lower in CKD (Figure 1(g)). At 48 h, the levels of SCFAs increased; the level of butyrate (Figure 1(g,h)) and acetate (Figure 1(i,j)) was not significantly different in both CKD patients and HV (without AA supplementation) but a significantly higher level of propionate was found (Figure 1(k,l)). The production capacity of both CKD patients and HV (without AA supplementation) was similar for butyrate, acetate, and propionate, as reflected by the delta (0–48 h; Figure 1(h,j,l)). During the 48 h incubation period, BCFA levels, generated through proteolytic fermentation of branched-chain AAs, rose (except isocaproate) in the incubated feces of CKD patients compared to HV (Figure 1(m-r)).

Next, we investigated the ability of the gut microbiota of CKD patients to generate precursors of p-cresyl sulfate, p-cresyl glucuronide, indoxyl sulfate, and TMAO (i.e., p-cresol, indoles, and trimethylamine (TMA), respectively)^32^ in both basal conditions and after the addition of the AAs mixture compared to HV (Figure 1(s-z)). In the basal condition (without AA supplementation), only the level of p-Cresol was significantly higher after 48 h of incubation in CKD compare to HV (Figure 1(s)). However, after spiking with AAs, the level of indole (but not IAA) and p-cresol was significantly higher after 48 h of incubation (Figure 1(s,u,w)). While the supplementation of choline and carnitine increased TMA levels, it is noteworthy that there was no significant difference in TMA concentration between CKD and HV (Figure 1(y)).

Collectively, these results illustrate alterations in the gut microbiota metabolism of CKD patients, showing a noticeably higher protein and AA catabolism, resulting in higher indole and p-cresol generation.

### Selection of the optimized multi-biotic formulation to target gut microbiota-derived metabolite generation

We next hypothesized that a specifically designed multi-biotic targeting *de novo* UTs generation may improve kidney function. The enzyme profiles of several industrially available probiotic strains were analyzed with a focus on UT biosynthetic pathways (Supplementary Figure S3a and Table S2). *Bifidobacterium animalis subsp. lactis* BB-12, *Bifidobacterium longum subsp. longum* NCC 2705, *Lacticaseibacillus rhamnosus* GG and *Lactobacillus johnsonii* NCC 533 did not harbor enzymes catalyzing the generation of either ammonia, TMA, IAA, indoles or their precursors. However, three of the strains harbored several enzymes catalyzing the generation of 4-hydroxyphenylpyruvate *(L. rhamnosus* GG = 4 enzymes, B. *longum* NCC 2705 = 6 enzymes and *B. lactis* BB-12 = 7 enzymes), which is a precursor of p-cresol (Supplementary Table S3). *L. johnsonii* NCC 533 therefore appeared to be the only strain devoid of the capacity to generate any of the above-mentioned UTs (Supplementary Figure S3b).

The genes of *L. johnsonii* NCC 533 induced during *in vivo* growth in the gut have been identified in a previous report.^33^ Particularly, different sugar phosphotransferase system and sugar transporters were found to be induced *in vivo*, including a predicted to be a cellobiose transporter.^33^ Furthermore, cellobiose was previously shown to enhance the adhesion capacity of *Lactobacillus acidophilus* NCFM belonging to a closely related species.^34^ Overall, this suggests that cello-oligosaccharides may be interesting to investigate for the promotion of the persistence of *L. johnsonii* NCC 533 in the gut. In the present study *L. johnsonii* NCC 533 was able to consume both cellobiose and cellotriose, to a similar extend as glucose. The strain also exhibited a high growth rate on all these substrates (1.4, 1.2 and 1.2 h^−1^, respectively). The strain was also able to grow on cellotetralose, but there was a longer lag phase and a lower growth rate (0.7 h^−1^) on this substrate, despite a higher final biomass (Supplementary Figure S3c). Overall, these results demonstrate that cellobiose is a suitable growth substrate for *L. johnsonii* NCC 533.

Finally, C4-C8 short-medium chain triglycerides, as a more palatable source of butyrate were added, given that we and others^5,10^ have observed significantly lower basal levels of butyrate in feces in CKD patients (Figure 1(g)). The selected multi-biotic for CKD, named SynCKD, was therefore defined as a mix of *L. johnsonii* NCC 533, cellobiose, and C4-C8 triglycerides; the dose selected was based on the safety of individual components in human studies (https://www.efsa.europa.eu/en).

### SynCKD reduces UT precursor generation ex vivo

**Figure 2.**
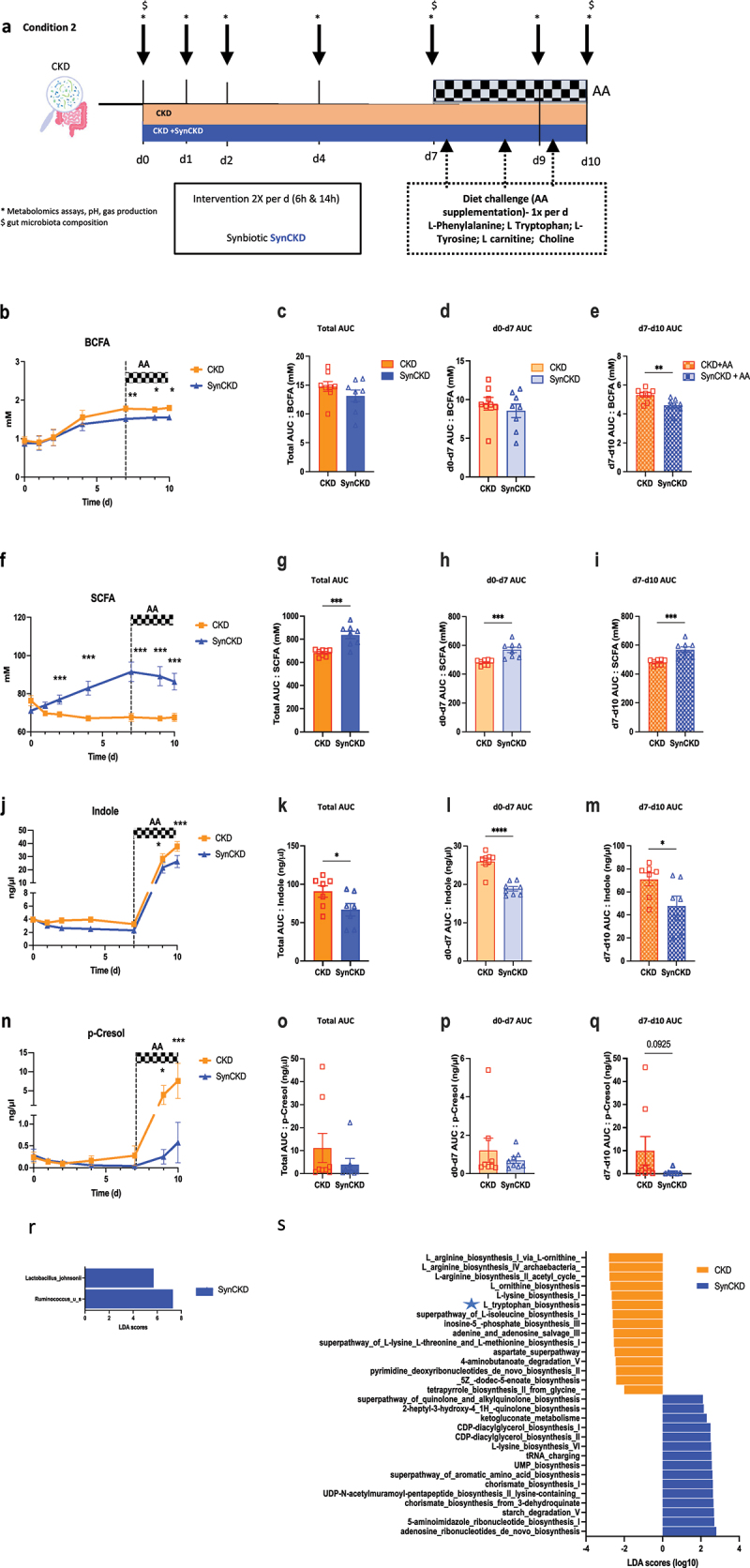
SynCKD decreased indole, phenol and branched-chain fatty acids (BCFA) generation and increased short-chain fatty acid (SCFAs) generation in the simulator of human intestinal microbial Ecosystem (SHIME) system colonized by feces from chronic kidney disease (CKD) patients during 10 days with or without amino acid (AA) supplementation in the diet challenge.

Next, in the condition 2, we evaluated this proprietary formulation intended to improve uremic dysbiosis and UT production using the SHIME colonized with feces from CKD patients (Figure 2(a)). First, before treatment, no significant difference was detected in microbiota composition across donors in control condition and with SynCKD (Supplementary Table S4 and S5). Adding AAs had no significant effect on the gut microbiota composition (Supplementary Table S4 and S5), and minor effect function (Figure S4a). We observed that SynCKD administration enhanced bacterial fermentation parameters, including the base–acid ratio and lactate (Supplementary Figure S4b-i). Interestingly, SynCKD was effective in, reducing urea and ammonium levels (Supplementary Figure S4j-q), and mitigating proteolytic activity (measured by BCFA generation) throughout all the incubation period (Figure 2(b-e)). As expected, SynCKD increased butyrate given that it contains C4-C8 triglycerides but also increased the production of other SCFAs. (Figure 2(f-i) and Supplementary Figure S5a-l). In addition, this formulation significantly reduced the generation of TMA and indole as reflected by the delta D0-D7 (Supplementary Figure S5o and Figure 2(l)), and counteracted the overgeneration of indoles and p-cresol induced by AA supplementation (Figure 2(j,k,m-o,q)). The other AA metabolites were not strongly modified by the treatment (Supplementary Table S6). Besides, the probiotic agent (*L. johnsonii*), SynCKD treatment stimulated the abundance of an unknown *Ruminococcus species* (Figure 2(r) and Supplementary Figure S5q) and influenced gut metabolic pathways, in particular the tryptophan biosynthesis (Figure 2s).

(a) Chronogram of the experiment (condition 2). Orange is the control situation (Feces from CKD patients) and blue is treated situation (Feces form CKD patients with SynCKD). (b) Branched-chain fatty acids (BCFA), (f) SCFAs (the sum of acetate, propionate, and butyrate) (j) indole, and (n) p-cresol generation and their respective total area under curve (AUC) (c, g, k, o), day 0-7 AUC (d, h, i, p), and day 7–10 AUC (e, i, m, q); *n* = 8 per group. In b, f, j, and n, the dotted line indicates initiation of AA supplementation diet-challenge. Data are mean ± standard error of the mean * *p* < 0.05, ** *p* < 0.01, *** *p* < 0.001 and **** *p* < 0.0001, respectively, for CKD *versus* SynCKD. Two-way ANOVA with Bonferroni *post hoc* test was performed for kinetic and one-way ANOVA with Bonferroni *post hoc* test for AUC. (r) The linear discriminant analysis effect size (LEfSe) bar plot showing significantly different abundant bacterial species between CKD *versus* SynCKD, ordered according to effect size, in samples collected on day 10. All pathways shown meet *p* ≤ 0.05 for Kruskal-Wallis and Wilcoxon tests, and have a Linear Discriminant Analysis (LDA) score ≥ 2.0 or ≤ -2.0; (*n* = 8 per group). (s) LEfSe bar plot showing significantly different enriched MetaCyc pathways between SynCKD and CKD, ordered according to effect size, in samples collected on day 10. All pathways shown meet *p* ≤ 0.05 for Kruskal-Wallis and Wilcoxon tests, and have an LDA score ≥ 2.0 or ≤ -2.0; (*n* = 8 per group).

### SynCKD reduces circulating UTs in CKD mice

**Figure 3.**
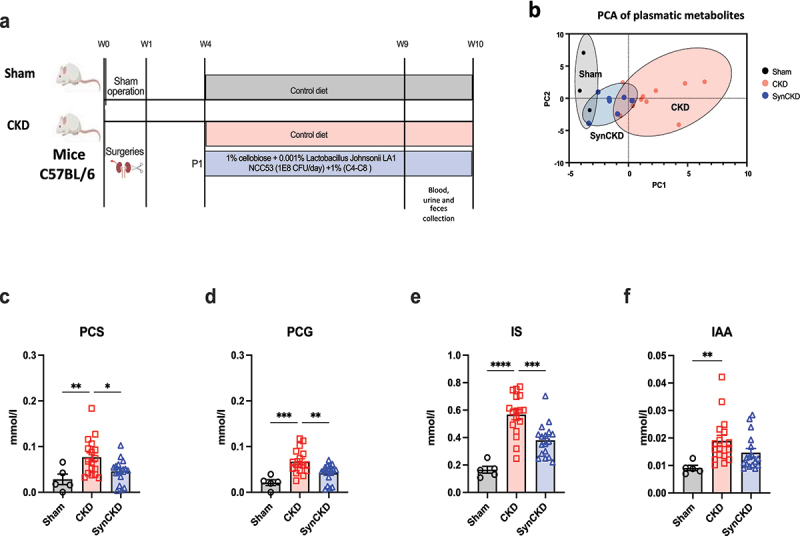
SynCKD reduced plasmatic uremic toxins in chronic kidney disease (CKD) mice.

Having established the potential beneficial properties of this multi-biotic *ex vivo*, we then tested SynCKD in a mouse model of CKD induced by 5/6 nephrectomy (Figure 3(a)). Principal component analysis (PCA) underscored the substantial differences in metabolite profiles after 6 weeks of treatment (Figure 3b), especially reducing the plasma levels of UTs such as PCS, PCG, and IS, compared to the sham group (Figure 3(c-e)). The concentration of IAA and dimethylarginine was not significantly affected by the SynCKD (Figure 3(f) and Supplementary Figure S6a). No significant effect of SynCKD was observed on TMAO, or its precursors, as well as the plasma levels of AAs (Supplementary Figure S6b-e).

(a) Chronogram of the experiment. (b) Separation of blood metabolites revealed by principal component analysis (PCA). The spatial density distribution of samples in each group is indicated with an ellipse. The x- and y-axis labels indicate the metabolic compositional variance explained by the first two principal components (*n* = 3–11). Concentration of microbiota-derived uremic toxins levels in the plasma (c) p-cresyl sulfate (PCS), (d) p-cresyl glucuronide (PCG), (e) indoxyl sulfate (IS), and (f) indole-3-acetic acid (IAA); *n* = 5–17. Data are mean ± standard error of the mean * *p* < 0.05, **p < 0.01, ***p < 0.001 and ****p < 0.0001 correspond to significant differences between groups. One-way ANOVA with Bonferroni *post hoc* test was performed.

### SynCKD improves kidney function in CKD rodent models

**Figure 4.**
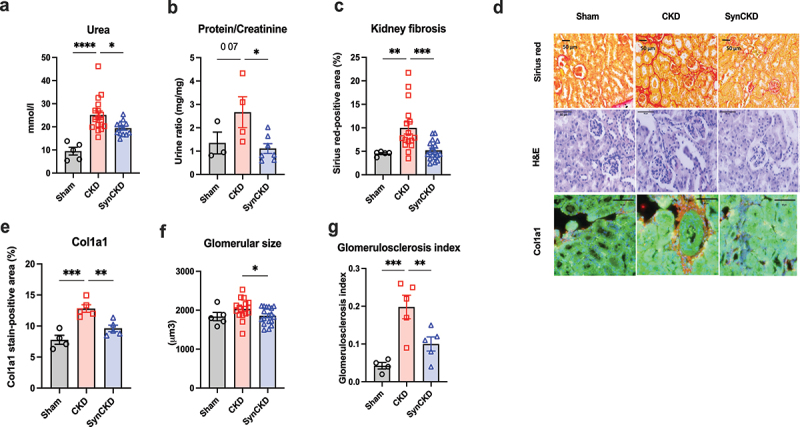
SynCKD improved kidney dysfunction in chronic kidney disease (CKD) mice.

As expected, CKD mice exhibited elevated plasma urea and proteinuria levels, which were significantly reduced by the treatment with SynCKD (Figure 4(a,b)). Furthermore, histological analyses revealed reduced kidney fibrosis in SynCKD-treated mice compared to the CKD group, as measured by Sirius red staining and by Col1a1 staining (Figure 4(c-e)). There was also a decrease glomerular hypertrophy and in glomerulosclerosis in CKD mice treated with the SynCKD (Figure 4(d,f,g)). To confirm the kidney-protective effect of SynCKD in other animal facility environments and across different species, we treated 5/6 nephrectomized rats with SynCKD or with lisinopril, an angiotensin-converting enzyme inhibitor (ACEi) used as a standard care for nephroprotection, for a duration of 8 weeks (Supplementary Figure S7a). We confirmed that the SynCKD was also able to reduce plasmatic UT concentration (Supplementary Figure S7b-e). Although neither SynCKD nor the ACEi treatment improved the glomerular filtration rate or plasma creatinine levels (Supplementary Figure S8a,b), we confirmed a positive impact of SynCKD, similar to that of lisinipril, on the ability to reduce albuminuria, kidney fibrosis and kidney inflammation (Supplementary Figure S8c-h).

Levels of (a) blood urea (*n* = 5–17) and (b) urine proteinuria/creatinine ratio (*n* = 3–5; for urine, mice were housed in pairs). (c) Quantitative analysis of the fibrotic area (Sirius red-positive area). (d) Sirius red staining of the kidney interstitium; Hematoxylin and Eosin (H&E) staining and Col1a1 immunofluorescence staining (scale bar = 50 μm). (e) Quantitative analysis of Col1a1 staining. (f) Quantitative analysis of glomerular size. (g) Quantitative analysis of glomerulosclerosis index. Data are mean ± standard error of the mean. * *p* < 0.05, ** *p* < 0.01, *** *p* < 0.001 and **** *p* < 0.0001 correspond to significant differences between groups. One-way ANOVA with Bonferroni *post hoc* test was performed.

To determine the specific impact of each compound, we treated CKD rats with SynCKD, *L. johnsonii* NCC 533 + cellobiose or C4-C8 triglycerides (Figure S9a). Interestingly, only SynCKD was able to significantly decrease both albuminuria (Figure S9c-e) and UT concentrations (Figure S9f-i) compared to *L. johnsonii* NCC 533 + cellobiose and C4-C8 triglycerides, alone.

### SynCKD mitigates gut microbial dysbiosis by reducing aromatic AA-metabolizing microbes in CKD mice

In order to understand the impact of SynCKD on gut microbiota, metagenomic analyses were performed on feces of CKD mice treated for 6 weeks. Approximately 20 million reads per fecal sample were generated, with a minimum of 17.0 million read pairs (Supplementary Figure S10). About 8.3 million read pairs per sample mapped to the gene catalog, constituting a mean 75.8% of high-quality nonhost (HQNH) reads and a minimum of 68.6% (Supplementary Table S7). Sequencing read quality control and gene catalog mapping statistics for each sample are presented in the Supplementary Table S8. The genes were annotated to 9503 KEGG orthology (KO), organized into 878 metagenomic species (MGSs; Supplementary Tables S9 and S10).

**Figure 5.**
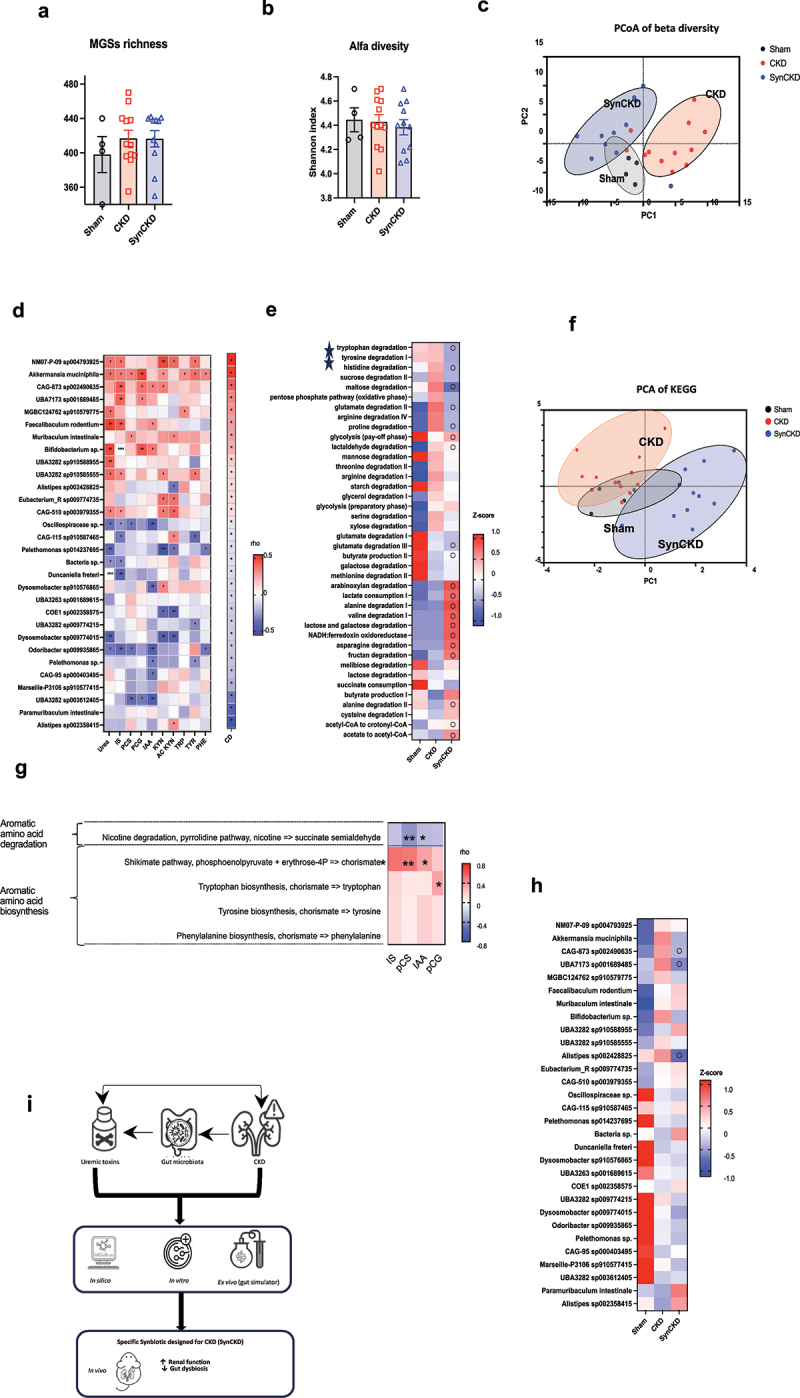
SynCKD influenced gut microbiota composition in chronic kidney disease (CKD) mice.

Compared to sham mice, CKD mice exhibited significant variations in microbial diversity, taxonomic composition and functional potential, indicative of CKD-associated microbiome alterations (Figure 5). MGS richness and alpha diversity (Shannon index) were similar across all groups (Figure 5(a,b)) but a significant difference in Bray-Curtis dissimilarities was observed among all groups (PERMANOVA; *p* < 0.0001) with a negligible cage effect (PERMANOVA; *p* = 0.17). Principal coordinate analysis (PCoA) underscored substantial differences in gut microbiome signatures between the Sham and CKD groups, with SynCKD-treated CKD mice showing gut microbiome signatures closely resembling the Sham group (Figure 5(c)). At the genus and family levels, further analysis found pronounced shifts in gut microbial community structure in CKD mice (Supplementary Figure S11a,b).

(a) Metagenomic species (MGSs) richness and (b) alpha diversity (Shannon index). All groups were compared pairwise using the Mann-Whitney U test. (c) Principal coordinates analysis (PCoA) based on Bray – Curtis dissimilarities among samples, calculated based on the MGS abundances. Samples and ellipses are color coded according to group. The x- and y-axis labels indicate the microbial compositional variance explained by the first two principal coordinates. (d) Heatmap showing the Spearman correlation coefficients between the concentration of uremic toxins (IS: indoxyl sulfate, PCS: p-cresyl sulfate, PCG: p-cresyl glucuronide, IAA: indole acetic acid, KYN: kynurenine, Ac KYN: kynurenic acid), their precursors (TRP: tryptophan, TYR: tyrosine, PHE: phenylalanine), urea and the most contrasted (cliff delta, CD) metagenomic species (MGSs) in Sham and CKD mice (*n* = 4–12). *p < 0.05, **p < 0.01, ***p < 0.001 and **** *p* < 0.0001 (e) Heat map showing the mean abundance of gut metabolic modules (GMMs) expressed by the Z-score (relative ratio of abundance) in Sham, CKD mice and CKD mice treated with SynCKD (*n* = 4–12). False discovery rates (q-value) were estimated using the Mann-Whitney test and the Benjamini-Hochberg procedure. Circles indicate *q* < 0.1 *versus* CKD group. (f) Principal component analysis (PCA) of the relative abundance of Kyoto Encyclopedia of Genes and Genomes (KEGG) modules. The spatial density distribution of samples in each group is indicated with an ellipse. The x- and y-axis labels indicate the microbial compositional variance explained by the first two principal components. (g) Heatmap showing the Spearman correlation coefficients between microbial functional modules and UTs. * *p* < 0.05 and ** *p* < 0.01. (h) Heat map showing the abundance MGSs expressed by the Z-score (relative ratio of abundance) in sham, CKD mice and CKD mice treated with SynCKD. False discovery rates (q-value) were estimated using Mann-Whitney test and the Benjamini-Hochberg procedure. Circles indicate *q* < 0.1 *versus* CKD group. (i) Summary of the study.

To further explore the links between gut microbiota and the generation of UTs, we carried out analysis of abundances of gut MGSs and plasma UT levels. There were significant variations between Sham and CKD mice for several MGSs (*n* = 64, ~6% of the total microbiome; Supplementary Table S11-S13). However, among the most contrasting MGSs between Sham and CKD mice, the most abundant in the latter were positively correlated with aromatic AA-derived UTs; the most abundant in Sham mice were negatively correlated with these UTs (Figure 5(d)). The CKD mice presented a reduction in gut metabolic modules (GMMs) responsible for SCFAs generation, but an enrichment in those involved in aromatic AA degradation, such as tryptophan and tyrosine degradation (Figure 5(e) and supplementary Figure S11c). PCA of the relative abundance of KEGG modules indicated a shift in CKD mice compared to Sham mice (Figure 5(f)), with several functional changes significantly correlating with plasma concentrations of aromatic AA-derived UTs (Figure 5(g)). These data support the hypothesis that elevated UTs in CKD mice are mainly driven by gut microbiome-mediated AAs.

The SynCKD led to a decrease in the mean abundance of several MGSs associated with high levels of UTs (Figure 5(h) and supplementary Figure S11d), as well as a decrease in GMMs involved in AAs degradation (Figure 5(e)). At the gene level, CKD mice had a higher expression of those coding for tryptophanase, urease, trysosinase, and lower expression of those involved in SCFAs generation, but SynCKD administration significantly counteracted tryptophanase and tyrosinase abundance (Supplementary Figure S12 and S13). Given that there was a positive correlation between gut-microbiota tryptophanase and IS (Supplementary Figure S12l) and 4-hydroxyphenylacetate decarboxylase (a key enzyme involved in p-cresol generation) and PCS (Supplementary Figure S12i), we can conclude that the modification of gut microbiota by SynCKD directly decreased plasmatic UTs.

## Discussion

This study aimed to determine whether a specifically designed multi-biotic may improve kidney function by reducing gut-derived UTs and increasing SCFAs generation. We first verified that uremic gut microbiota played a direct role in the over-generation of UTs using a gut simulator inoculated with feces from CKD patients. Then, using this approach, we selected an optimized multi-biotic able to reduce UT generation *ex vivo* and *in vivo* in two different CKD rodent models. Moreover, through shotgun metagenomic sequencing, we demonstrated that the selected multi-biotic had a beneficial impact on microbiota composition. In particular, the multi-biotic reduced the abundance of microbial genes involved in tryptophan and tyrosine degradation resulting in the reduction of UTs, such as indole- and phenol-derived accumulation in CKD mice. Last but not least, the selected multi-biotic slowed down CKD progression in two different pre-clinical models of CKD in terms of both geographical location and species. (Figure 5i).

The association between gut dysbiosis and CKD has been extensively studied in both rodent models and human.^3^ Most studies confirm altered gut microbiota composition along with CKD progression. Metagenomic data also found an increase in bacterial genes involved in UT generation in CKD patients.^17^ However, these findings only establish associations, not causality, and the activity of these genes must be explored, as post-translational modifications may influence function. For example, Lobel et al. demonstrated that an increase in the consumption of sulfur-containing AAs induces post-translational modifications of bacterial tryptophanase leading to a decrease in the capacity for indole generation.^35^ Fecal transplantation experiments further support the toxic role of uremic microbiota,^17,36^ with transplanted CKD mice showing higher UT levels and reduced kidney function,^17^ while feces from sham mice lowered plasma UTs.^37^ Deciphering UT production by the microbiota is complex due to the interaction between kidney function and intestinal barrier leakage. Using anaerobic cultures of fecal samples, Gryp et al. did not observe a difference in *ex vivo* p-cresol, indole, and IAA generation.^15^ Herein, we used a gut simulator, which replicates the physicochemical conditions of the intestine, allowing an optimal survival of intestinal bacteria. We observed urea consumption alongside increased ammonium production, suggesting enhanced bacterial urease activity in CKD.^4,38^ AA supplementation attenuates this difference, likely by activating alternative bacterial metabolic pathways, but further studies are needed to confirm this mechanism. Additionally, we found that the over-generation of UT precursors was more significant after an AA challenge simulating a meal. This aligns with findings that lower protein diets reduce UT levels in CKD patients.^39,40^ Interestingly, no over-generation of TMA (the precursor of TMAO) was observed, consistent with recent studies showing kidney function as the main predictor of circulating TMAO levels.^41^ Overall, we demonstrated that the microbiota is certainly a key player in the generation of UTs and remains an interesting therapeutic target. The limitations of the present study are that we focused on a few metabolites of interest (cresol, indole, TMA, SCFAs) and that we included CKD patients older than HVs and treated with several drugs that could themselves influence the gut microbiota. Future studies with an untargeted approach would be interesting to potentially identify other metabolic pathways of interest (e.g., bile acids, kynurenine pathway, *etc*.).

Multiple strategies have been attempted to target gut microbiota or its metabolites for CKD management, but success has been limited. Studies have consistently shown that the survival, safety, and efficacy of probiotic candidates are strain-specific and cannot be generalized.^18–21^ As a result, regulatory agencies have established guidelines for evaluating new probiotic/symbiotic/multi-biotic candidates, including *in silico*, *ex vivo*, and *in vivo* studies, with genomics as a key tool for rapid screening.^42^ Indeed, other potential probiotic strains may have characteristics similar to *L. johnsonii* NCC533, as we have not screened or studied the entire range of available probiotic strains. For the first time, we applied this workflow in CKD to select an appropriate multi-biotic. Other approaches have also been used, such as the finding by Li et al. of *Faecalibacterium prausnitzii* depletion in CKD patients and its improvement of kidney function with supplementation.^5^ Zhu et al. selected *L. casei Zhang* for its ability to modulate the immune response, demonstrating its capacity to elevate SCFAs and improve kidney function in CKD.^6^ Overall, these data confirm the importance of carefully selecting probiotics to improve kidney function.

*L. johnsonii* NCC 533 is part of the acidophilus group of intestinal lactobacilli, and its probiotic potential has been extensively explored.^43–45^ *In silico*, *L. johnsonii* NCC 533 exhibits several favorable properties, including pathogen inhibition, epithelial cell attachment, and immunomodulation; furthermore, it lacks genes for AAs biosynthesis and urease, making it unlikely to produce UTs or ammonium.^43^ While its role in health and disease is unclear, evidence links its low abundance to unhealthy conditions. A Western diet decreases *L. johnsonii* in healthy humans,^46^ and in CKD mouse models, its abundance was also reduced,^47^ though no data exist for CKD patients. In the present study, *L. johnsonii* was not detected in mice. Preclinical data suggested that *L. johnsonii BS15* may provide kidney protection in mice with acute kidney injury.^48^ More recently, *L. johnsonii* NCC 533 has been identified as acting on liver mitochondria, improving lipid metabolism in T2D^46^ and alleviating acute myocardial infarction.^49^ In the CKD rodent models used in the present study, no significant impact on lipid parameters or glucose tolerance was observed, though these changes are mild in CKD.

Evidence suggest that some probiotics exert anti-inflammatory effects by increasing SCFA levels. Butyrate positively impacts kidney function by suppressing inflammation through kidney butyrate-GPR43 signaling.^50^ A recent study found lower plasma and fecal SCFA levels in CKD patients,^51^ and we confirmed reduced basal butyrate levels in CKD patients feces. However, although the gut microbiota of CKD mice was depleted in genes involved in SCFAs generation herein, we did not observe a decrease of this *ex vivo* in an artificial intestine by the gut microbiota from CKD patients. This suggests the capacity for SCFA generation in CKD patients microbiota remains intact but requires further study. Given the role of butyrate in CKD progression, even a small reduction may affect gut function and structure. Butyrate administration in CKD rodent models has delayed disease progression, but concentrations used were much higher than in the present study.^5,12,51^ Unlike sodium butyrate used previously, we included a C4-C8 triglyceride formulation providing butyric acid in a more palatable form suitable for human use.

Interestingly, *in vitro*, SynCKD specifically increased the abundance of R*uminococcus*. Several studies on CKD patients have observed *Ruminococcus* less abondnat and a good discriminator between CKD patients and HV,^52^ even though these studies seem contradictory.^16,52^ Additionally, *Ruminococcus* is positively associated with high lupus nephritis activity.^53^ However, in other metabolic study, its concentration was significantly lower in non-obese Metabolic Associated Fatty Liver Disease (MAFLD) subjects compared to controls, and a role in bile acids metabolism has been suggested.^54^ Therefore, the specific role of R*uminococcus* in kidney disease warrants further investigation.

The present study has several limitations. Firstly, we did not test each ingredient individually for UT generation *in vitro*. However, we investigated the specific impact of each compound *in vivo*, and found that only SynCKD limited albuminuria and UT accumulation, suggesting a synergic effect. PCS and IS levels are associated with different gut microbiota compositions, indicating that a single intervention may not be sufficient to target both UTs.^14^ This present study confirms this, as *Lactobacillus johnsonii + cellulose* reduced PCS but not IS, unlike C4-C8, underscoring the need for different products to modulate distinct microbiota profiles. Moreover, this study aimed to translate findings rapidly to CKD patients, using doses approved for humans; therefore, so different dose-response relationships were not explored and ensuring the absence of toxicity remains challenging and warrants further investigation in clinical settings. Secondly, the mechanism that explains the beneficial effect of SynCKD (and the individual components) needs to be further clarified. While we observed a reduction in circulating uremic toxins, it is unclear whether this effect is primarily driven by improved kidney function, modulation of the gut microbiota, enhanced intestinal barrier integrity, or host metabolic reprogramming. The mechanistic hypothesis is supported by the shotgun microbiota analysis and the *in vitro* model because the administration of SynCKD profoundly modifies the function of the microbiota and consequently the production of UT. As no combinations improved measured glomerular filtration rate in CKD rats, this suggest the effects are primarily due to microbiota changes. Thirdly, UT levels were not measured in the gut, but recent reports show a strong correlation between intestinal and blood UT levels.^14,15,17^ Another key issue is the translatability of kidney protection from rodents to humans. Differences in gut microbiota between species are significant due to intrinsic factors, though overall patterns may be similar.^55^ Given that our primary objective is to reduce UT production, we aim to decrease the overall toxin-producing species.^14,17^ We have confirmed that SynCKD influences UT production pathways in both humans and mice, while acknowledging that additional mechanisms and specific rodent pathways could further improve renal function. Finally, the number of patients included was limited, and we only selected non-diabetic individuals to minimize the confounding effects of diabetes and obesity on microbiota function. As such, the results cannot be generalized to the broader CKD population. Further studies in larger and more diverse cohorts are warranted.

In summary, the present study confirms that alterations in gut microbiota composition and function contribute to play a crucial role in the progression of CKD and UT generation. Although recent advancements CKD treatment, such as the introduction of sodium-glucose cotransporter-2 inhibitors and glucagon-like peptide-1 agonists, have shown improvements, some patients still experience a persistent decline in renal function. Modulating the microbiota presents a promising therapeutic target and appears safe to complement existing therapies and limit renal deterioration.^18^ Currently, no strategy targeting the microbiota has proven effective in improving renal function, as the selection of biotics has been largely empirical.^21^ Through a step-by-step approach, we designed a specific multi-biotic for CKD that slowed down the progression of CKD through the improvement of gut microbiota-derived metabolites. Our study encourages further consideration of biotic choices to increase the chances of success. However, these results must be confirmed through a clinical randomized controlled trial focused on long-term outcomes, such as the slope of GFR.

## Supplementary Material

supplementary_methods_finalR2_clean.docx

## Data Availability

The data that support the findings of this study are available from the corresponding author upon reasonable request. The shotgun metagenome sequence datasets obtained from this study have been deposited in the French repository at “Recherche.data.gouv.fr” and can be accessible with the following link: https://doi.org/10.57745/Q9L6ZH.
