# Supplementary material for: A specifically designed multi-biotic reduces uremic toxin generation and improves kidney function
Source: Gut Microbes. 2025 Jul 12;17(1):2531202. doi: 10.1080/19490976.2025.2531202 (PMC12258176; doi:10.1080/19490976.2025.2531202)
Supplement: supplementary_methods_finalR2_clean.docx [file KGMI_A_2531202_SM7577.docx]

**SUPPLEMENTAL FILES**

**Supplementary materials and methods**

***In silico* analysis**

**Uremic toxin synthesis pathways reconciliation**

The European Uremic Toxin Work Group has listed 90 compounds considered to be uremic toxins.^S1^ For our search, we focused specifically on urea, trimethylamine N-oxide (TMAO), indole-3-acetic acid (IAA), indoxyl sulfate (IS), and p-cresyl sulfate (PCS). Bacterial metabolic pathways for these uremic toxins were compiled starting from the Kyoto Encyclopedia of Genes and Genomes (KEGG) and complemented by a literature search.

***In silico* screening of industrially available probiotic strains**

Amino acid (AA) sequences of all predicted proteins from *Lactobacillus johnsonii* NCC 533 (*L. johnsonii* NCC 533), *Bifidobacterium animalis subsp. Lactis BB-12*, *Bifidobacterium longum subsp. Longum NCC 2705*, and *Lacticaseibacillus rhamnosus* GG were retrieved from the Refseq database (https://www.ncbi.nlm.nih.gov/refseq/) and compiled. The AA sequences of the enzymes for each reconciled pathway were extracted from UniProt (https://www.uniprot.org) according to the Enzyme Commission number (EC number) and compiled in single fasta files for each pathway. These fasta files containing the protein sequences of key enzymes were the reference sequences to perform Basic Local Alignment Search Tool for protein sequences (BLASTp) ^S2^ against the above mentioned protein sequences. A threshold of 80% coverage to the reference protein was applied and in case of low identity (< 30%), InterProScan^S3^ was further used to predict the presence of relevant functional domains.

**Growth profile of *Lactobacillus johnsonii* NCC 533 on cello-oligosaccharides**

*Lactobacillus. johnsonii* NCC 533 was obtained from the Nestlé Culture Collection (Nestlé Research, Lausanne, Switzerland) and reactivated in Man, de Rogosa & Sharpe medium (MRS)^S4^ using two subsequent growth passages (37°C, 24h each). Subsequent experiments used this fresh culture at a 2% inoculation rate. The inoculum was washed with 1 volume of sterile phosphate-buffered saline (PBS) to avoid carry-over of residual sugars. Culturing was performed in a modified MRS medium lacking carbohydrates (MRS-C)^S5^ to which cello-oligosaccharide (cellotriose, cellobiose, or cellotetraose) or glucose was added at a final concentration of 1% (w/v). Cello-oligosaccharides were obtained from Megazyme (Wicklow, Ireland) and glucose from Sigma-Aldrich (Saint-Quentin Fallavier, France). A 4% (w/v) solution of each carbohydrate was prepared and filter-sterilized through a 0.22μm filter. The growth was performed in a BioLector microbioreactor (m2p-labs, Baesweiler, Germany). Each run was tested in a 48-well flowerplate (m2p-labs, Baesweiler, Germany) with 1ml volume per well. Plates were incubated anaerobically with a CO_2_ gas phase and shaking at 300rpm for 48h. Biomass and pH changes of each well were recorded over the incubation period.

**Multi-biotic composition**

Doses of each component were optimized based on the safety of individual components and their use in human studies (https://www.efsa.europa.eu/en). We then applied allometric scaling to define the corresponding doses for *in vitro* and *in vivo* studies.

***In vitro* analysis**

**Collection of fecal samples from healthy volunteers and CKD donors**

Ten non-obese chronic kidney disease (CKD) patients and 10 healthy volunteers (HV) were recruited (NCT04768309, CPP Sud EST II, N° 202-007). The selection criteria were: ability to provide fresh feces at a particular time, and, for CKD patients an estimated glomerular filtration rate (eGFR) < 30 ml/min/1.73m^2^, without diabetes, inflammatory disease, obesity (Body Mass Index (BMI) between 18-30 kg/m2), as well as no antibiotic, laxative, probiotic or prebiotic during the previous month), and, for HV, eGFR ≥ 90 ml/min/1.73m^2^, no albuminuria, no treatment, and no history of kidney injury. The diet profile was quantified using the last 3-day recall. The amount of macronutrients (protein, lipids, carbohydrates, and fibers) as well as calories from the dietary inquiries were analyzed using the French food composition table – CIQUAL (the French agency for food, environmental and occupational health safety).

To minimize oxygen exposure during transportation (from the patient’s home to aliquoting at ProDigest, [Gent, Belgium]), after bowel movement, the fecal samples were immediately transferred into a container under anaerobic conditions using an airtight box equipped with a deoxidizer (Thermo Scientific™ Oxoid AnaeroGen, Fisher Scientific, Illkirch, France). They were then kept at 4°C before being sent to ProDigest at 4°C < 24h. However, due to packaging issues (e.g., improperly sealed boxes) and deviations from cold-chain preservation requirements, several samples did not meet quality control criteria. Upon arrival the stool samples of 9 HV and 8 CKD patients were usable. At the reception, a fecal slurry was prepared by homogenising fecal material in an anaerobic phosphate buffer (15 % w/v), composed of 8.8 g/L K2HPO4, 6.8 g/L KH2PO4, 0.1 g/L sodium thioglycolate, and 0.015 g/L sodium dithionite in pure water with pH adjusted to 7.0. Separate fecal slurries were prepared for each donor. After removing particulate material by centrifugation (2 min at 500 g), the fecal slurry was diluted (50:50) with an in-house cryoprotectant (42% w/v glycerol, 5 % w/v of DMSO, 0.5 g/L cysteine HCl, 10 g/L trehalose and 3 g/L tryptic soy broth) as previously described by Hoefman et al.^S6^ Each fecal slurry was aliquoted for the different project phases, flash frozen and then preserved at -80°C. Just before the experiment, an aliquot was defrosted and immediately added to the reactors. Use of aliquoted cryostocks of a single fecal suspension ensures that identical microbial communities are obtained in each aliquot, and thus that an identical inoculum is used throughout the different project phases. Moreover, preservation of aliquots from the start ensures that each aliquot undergoes only one freeze-thawing cycle before being introduced into the reactors. These actions ensure optimal reproducibility.

**Laboratory measurements**

For HV and CKD patients, all laboratory data were measured at the baseline visit, in a fasting state and stored at -80°C for future use. Biochemical parameter measurements were made using standard methods in the routine clinical laboratory. Creatinine was assayed using an enzymatic method (Roche Diagnostics, Meylan, France).

**DNA extraction, 16S rRNA sequencing and data analyses in CKD and HV before in vitro study**

Analysis of the intestinal microbiota of HV and CKD patients before the preparation with cryoprotectant was carried out by sequencing bacterial 16S rRNA by the ProfilExpert platform (Lyon, France). Purified DNA were extracted from 15 mg of feces with the Zymobiomics DNA microprep kit (Zymo Research, ref #D4301). 40 ng of DNA were used for the generation of libraries targeting the V3-V4 regions (Quick-16S NGS Library Prep Kit, Ozyme) and then sequenced on Illumina MiSeq standard v3 (New England Biolabs, Évry-Courcouronnes, France). The sequences were “demultiplexed” using Bcl2fastq software (v2.17.1.14) then cleaned with cutadapt (v1.9.1). Amplicon sequence variants were identified using Qiime2 DADA2 with a Zero noise OTU approach. Finally, a taxonomic assignment was made with Greengenes-13.8-nr99 base.

**SHIME**

The Simulator of the Human Intestinal Microbial Ecosystem (SHIME) system was first described by Molly *et al*.^S7^ and details of the SHIME set up have been published^S8^ (ProDigest). It consists of a succession of five reactors simulating the different parts of the human gastrointestinal tract (Fig. 1a). The first two reactors are of the fill-and-draw principle to simulate different steps in food uptake and digestion, with peristaltic pumps adding a defined amount of SHIME feed (140 ml 3x/day; 1 g l^−1^ arabinogalactan, 2 g l^−1^ pectin, 1 g l^−1^ xylan, 3 g l^−1^ potato starch, 0.4 g l^−1^ glucose, 3 g l^−1^yeast extract, 1 g l^−1^ pepton, 4 g l^−1^ mucin, 0.5 g l^−1^ cystein) and pancreatic and bile liquid (60 ml 3x/day), respectively to the stomach and small intestine compartment and emptying the respective reactors after specified intervals. The last three compartments simulate the large intestine. These reactors are continuously stirred, they have a constant volume and pH control. Retention time and pH of the different vessels are chosen to resemble *in vivo* conditions in the different parts of the colon. Upon inoculation with fecal microbiota, these reactors simulate the ascending, transverse, and descending colon. Inoculum preparation, retention time, pH, temperature settings, and reactor feed composition have been described by Possemiers *et al*.^S9^ Upon stabilization of the microbial community in the different regions of the colon, a representative microbial community is established in the three colon compartments, which differs both in composition and functionality in the different colon regions. To optimally address the impact of SynCKD in the present study, a customized reduced SHIME set-up was used, combining the upper gastrointestinal tract with a single colonic region. The colonic region simulated the transverse colon conditions, having a pH of 6.2 to 6.6 and a retention time of 32h. In this way, a diverse microbial community can be maintained in the system, being able to perform both saccharolytic as well as proteolytic fermentation processes. As CKD patients were used as donors, which are believed to have a dysbiosis, a reduced timeline was implemented in order to be able to maintain the dysbiosis. The SHIME experiment for this study consisted of two stages:

-Inoculation period: On the first day, the colon reactors were inoculated with an appropriate fecal sample. Then, 7 mL of the frozen stabilized microbiota of each of the investigated donors (which served as microbial source) was added, bringing the total volume in the reactors to 70 ml. Fecal microbiota was allowed to grow and colonize the reactor. After this overnight incubation, the colon reactors were fed with the basic nutritional matrix for 2 more days, to support the maximum diversity of the gut microbiota originally present in the fecal inoculum. This also allowed the microbial community to differentiate in the different reactors according to the local environmental conditions, while still retaining its CKD characteristics. Two conditions have been tested:

-Condition 1 (HV vs CKD): During 48h period, the SHIME reactor was operated under nominal conditions and fed 3 times per day with the SHIME nutritional medium with and without supplemented with an AA mix. On the first hour (0h) of this period, all arms were operated under nominal conditions, and samples collected at this time provided the baseline parameters. We have 4 groups: the control arm of each donor (CKD and HV) and the diet-challenging group of each donor supplemented additionally with an AA mix (CKD + AA and HV + AA). The AA mix was added during 1 of the feeding cycles (i.e., once a day; see Supplementary Table S1 for composition). The large volumes in the colonic regions allow the collection of sufficient volumes of liquids each day, without disturbing the microbial community or endangering the rest of the experiment. Samples were collected from the colon reactors at hours 0, 6, 12, 24, and 48 to investigate the concentration of metabolites, pH, and gas, as well as the resident microbial community composition (only at day 0).

-Condition 2 (CKD vs CKD + SynCKD): During 11-day period, the SHIME reactor was operated under nominal conditions and fed 3 times per day with the SHIME nutritional medium. On the first day (d0) of this period, all arms were operated under nominal conditions, and samples collected at this time provided the baseline parameters. The last 3-days, AA mix were added in the SHIME reactor. The AA mix was added during 1 of the feeding cycles (i.e., once a day). We have 2 groups: the control arm of each CKD donor (CKD) and the treatment arm (CKD + SynCKD). The addition of the test product in the treatment arm of each donor occurred during 2 of the feeding cycles (i.e., twice a day). Samples were collected from the colon reactors on days 0, 1, 2, 4, 7, 9, and 10 to investigate the concentration of metabolites, pH and gas as well as the resident microbial community composition (only at day 0; 7 and 10).

The composition of the SynCKD is:

1) Cellobiose at an *in vitro* dose of 2.625 g/d, representative of half of the *in vivo* dose, as the SHIME represents half a human. The dose of this prebiotic test product was reduced by 50% as to not overload the *in vitro* system. This dose was split over 2 feeding cycles;

2) Butyric acid and caprylic acid (C4-C8 triglycerides), at an *in vitro* dose of 62.5 mg/d and 162.5 mg/d, respectively, representative of the *in vivo* dose when considering absorption in the upper gastrointestinal tract and that the SHIME represents half a human. This dose was split over 2 feeding cycles;

3)The test probiotic *L. johnsonii* NCC 533 at a dose of 2*10^9^ CFU/day. This dose was split over 2 feeding cycles.

**Measurement of gas, pH, SCFAs, and ammonium**

Gas generation was measured with a pressure meter (Hand-held pressure indicator CPH6200; Wika, Echt, The Netherlands) and pH measurements were carried out with a Senseline pH meter F410 (ProSense, Oosterhout, The Netherlands). pH values were shown as the difference compared to the blank incubation. The mean ± standard deviation of pH of the blank at 0h, 24h, and 48h was respectively 6.61 ± 0.01, 6.40 ± 0.01, and 6.34 ± 0.01. Lactate was quantified using a commercially available kit, according to manufacturer’s instructions (R-Biopharm, Darmstadt, Germany). Short-chain fatty acid (SCFA; acetate, propionate, and butyrate) and branched-chain fatty acid levels (BCFA: isobutyrate, isovalerate, and isocaproate) were measured via a Gas Chromatography-Flame Ionization Detection (GC-FID) method described by De Weirdt *et al*.^S10^, after applying a diethyl ether extraction (with addition of 2-methyl hexanoic acid as internal standard). Ammonium was quantified via steam distillation, followed by titrimetric determination with hydrochloric acid (HCl).^S11^

**Targeted Metabolomic analysis**

**Analytical methodologies**

*In vitro* fluids and blood samples were subjected to a liquid extraction protocol based on the work of Vanden Bussche *et al.*^S12^ and De Paepe *et al.* ^S13^ Briefly, samples were vortexed 30s at 400g at room temperature, and then centrifuged 5min (13300g at 4 °C). The resulting supernatant was passed through a polyvinylidene fluoride filter (13mm diameter, 0.22μm) into a 2ml microtubes. From this tube, 300μl of fluid were placed into a liquid chromatography vial and 600μl of ultrapure water (containing the internal standards) were added. A 5μl aliquot was injected into the Ultra-High Performance Liquid Chromatography-High Resolution Mass Spectrometry (UHPLC-HRMS) system for polar metabolomic analysis. Polar metabolomic instrumental analysis was performed according to Vanden Bussche *et al.*^S12^ and De Paepe *et al.*^S13^ Chromatographic separation was achieved on a Vanquish quaternary pumping system (Thermo Fisher Scientific, Waltham, MA, US), equipped with an Acquity HSS T3 C18 UHPLC column (1.8μm, 150 x 2.1mm; Waters Corporation, Guyancourt, France). A binary solvent system consisting of ultrapure water and acetonitrile, both acidified with 0.1% (w/v) formic acid, was used at a constant flow rate and by applying a gradient profile. Detection was performed on a Q-Exactive™ standalone bench top quadrupole-Orbitrap high-resolution mass spectrometer (Thermo Fisher Scientific) which was preceded by heated electrospray ionization in polarity switching mode. The instrument was operated at a resolution of 140,000 full width at half maximum and in full-scan mode (m/z scan range of 53.4-800Da).

**Quality assurance**

Prior to analysis, the Mass Spectrometre system was calibrated according to the manufacturer’s guidelines (Thermo Fisher Scientific) to warrant accurate mass measurements (< 5ppm mass deviation) in both positive and negative ionization mode. In addition, the chromatographic and mass spectrometric performances were evaluated by injecting standard mixtures containing the target metabolites as well as internal standards. Biological samples were analyzed in a randomized order across the various experimental conditions, however, kept together per donor. Quality control (QC) samples were used to condition the Liquid Chromatography-Mass Spectrometre (LC-MS) instrument as well as to perform continuous monitoring of the instrument performance by repeated analysis of the QC-sample. Hereby, two QC-sample types were considered (by extracting pooled material, from 8 different samples, enriched, or not, with the targeted metabolites prior to extraction).

**Identification and absolute quantification of metabolites**

A metabolite was positively identified in the LC-MS raw data taking into account the accurate m/z-value of the molecular ion (allowed mass deviation of 5ppm), the 13C isotope pattern, and the relative retention time (taking into account the retention time of the nearest eluting internal standard, allowed time deviation of 2.5%). For identification and quantification of metabolites, Xcalibur software (Thermo Fisher Scientific) was used. In general, a metabolite was considered below the limit of quantification (LOQ) if a metabolite’s observed peak area was below 250,000 arbitrary units. However, the general noise level, stability of retention time, and the coefficient of variance as determined for the repeated measure of QC-samples, were additional features to assess the reliability of detection and quantification. Following peak integration, the area ratio was determined for each metabolite by calculating the ratio between the area of the metabolite and the one of the most suited internal standard. In addition, data normalization based on the QC-samples was performed. Absolute quantification was based on eight-point (including blank) calibration.

**Analysis of the microbial community composition for the *in vitro* analysis**

Two techniques were combined according to Vandeputte *et al*. ^S14^ to map the community shifts induced by the different treatments in large detail:

- Deep shotgun sequencing, providing proportional abundances of different taxa with high resolution, up to the (sub)species level.

- Accurate quantification of total bacterial cells in the samples using flow cytometry.

By combining the high-resolution phylogenetic information of deep shotgun sequencing with accurate enumeration of cell counts via flow cytometry, quantitative abundances of the different taxonomic entities inside the reactors are obtained. Functional capabilities of the microbial community were described by the MetaCyc metabolic pathways.

***In vivo* analysis**

**Animals**

C57BL/6J male mice of four weeks of age (ENVIGO), were housed in an air-conditioned room with a controlled environment of 21 ± 0.5°C and 60-70% humidity with a 12h light/dark cycle. A total of 40 mice were allowed a one-week period of acclimatization with free access to food and water, and were randomly assigned to 1 of 7 cages (5-6 mice per cage). There are 3 groups: Sham group (cage 1; n=5); CKD group (cage 2 to 4, n=18) and SynCKD group (cage 5-7, n=17). We plan to use 15 animals per group in the CKD model to detect a 10% difference in fibrosis, based on our previous observations^S15^. Considering a mortality rate of 10% in our previous model, we included 18 mice per CKD group. Only 6 mice were included as controls. Mice in the CKD and SynCKD groups underwent a 5/6 nephrectomy to induce moderate CKD, as previously reported,^S15^ while the Sham group was subjected to a sham operation.

Briefly, mice were anesthetized with ketamine/xylazine 100/20 mg/kg. The upper and lower poles of the right kidney were resected by electro-coagulation to induce. One week later, the left kidney was removed after ligation of the kidney blood vessels and the ureter. Special care was taken to avoid damage to the adrenals. All mice were given buprenorphine (0.05mg/kg intraperitoneally, 3 times a day) for 2 days to prevent post-surgical pain.

Sham and CKD mice groups were feed with standard diet (A04 diet) during all the procedure. Three weeks after the second surgery, SynCKD received for 6 weeks the A04 SAFE diet supplemented by SynCKD (10^8^ *L. johnsonii* NCC 533*,* 1% cellobiose and 1% C4-C8 triglycerides).

Body weight was measured twice a week whereas food and water intake were recorded three times a week. Food spillage, evaluated in preliminary experiments, was lower than 5% and therefore considered to be negligible. All experimental procedures were performed in accordance with the guidelines laid down by the French Ministry of Agriculture (n°2013-118) and the European Union Council Directive for the protection of animals used for scientific purposes of September 22^nd^, 2010 (2010/63UE). The study protocol was approved by the local ethics committee (#LS_2020_004 et 2020_005 and APAFIS#30603-2021012517248777 v3). All animals were analyzed and no animal were excluding except in case of premature death (2 deaths, one in the sham group and one in the SynCKD group before treatment initiation.).

Sixty one adult male Wistar RjHan:WI rats (Janvier, Genest Saint Isle, France) were allowed a one-week period of acclimatization with free access to food and water and individually housed in cages. They were randomly assigned to 4 groups: sham (n= 12), CKD (n=16), SynCKD (n=18) and CKD LISINOPRIL (n=17). 76 rats adult male Wistar RjHan:WI rats were randomly assigned to 5 groups: sham (n= 10) , CKD (n=15), SynCKD (n=17), ProB (1% cellobiose + 0,001% Lactobacillus Johnsonii LA1 NCC53; n=15) and C4-C8 (1% C4-C8 triglycerides; N=16). The 5/6 nephrectomy was conducted in a two-step surgical procedure under isoflurane anesthesia in. rats The treatments were administered over 8 weeks, three weeks after the second surgery. The animal facility is a fully Association for Assessment and Accreditation of Laboratory Animal Care (AAALAC) accredited unit, and all animal experiments were conducted in accordance with local bioethical guidelines, which are fully compliant with internationally accepted principles for the care and use of laboratory animals. All experiments are licensed by the Danish Animal Experimentation Council. All animals were analyzed.

All experiments are conformed with the ARRIVE guidelines (see ARRIVE checklist).

**Diuresis and 24h proteinuria**

After 6 weeks of diets, mice in groups of 2 (and rats individually) were housed for 24h in metabolic cages (Charles River laboratories, Ecully, France), to collect 24h diuresis. Owing to time constraints for CKD mice only a limited number of pairs were investigated (n=2 pairs and 1 individual for Sham, n= 4 pairs for CKD and n= 6 pairs for SynCKD). Urine volume was determined gravimetrically, and protein concentration was measured according to the Bradford method (Bradford reagent; Sigma-Aldrich) using bovine serum albumin (BSA) as standard. Albumin was measured using a commercial Enzyme-Linked Immunosorbent Assay (ELISA) kit (Bethyl Laboratories, Montgomery, TX, US)

**Tissue and blood collection - biochemical measurements**

At the end of the experiment, animals were euthanized using ketamine/xylazine (100/20mg/kg), blood was collected by left ventricular puncture using a syringe containing 0.2μmol of EDTA and then centrifuged for 8 minutes at 10000g to separate plasma. Plasma samples were stored at -80°C. One kidney was stored for 48h in a 4% (w/v) paraformaldehyde solution for histology, and the second one was frozen in liquid nitrogen and kept at -80°C. Urea was measured by a commercial assay kit (Cayman, Ann Arbor, MI, US). Creatinine was measured using a commercial kit (Roche Diagnostics, Meylan, France).

**Transcutaneous measurements of Fluorescein Isothiocyanate (FITC)-Sinistrin clearance for Glomerular Filtration Rate (GFR) estimation in rats**

Under light isoflurane anesthesia, Medibeacon transdermal GFR monitors were mounted to the back of the animal according to manufacturer’s instructions. Baseline autofluorescence was measured for a minimum of 5min before a bolus of FITC-sinistrin (40 mg/ml, 0.075 mg/g BW) was injected into the tail vein. Plasma clearance of FITC-sinistrin was then monitored transcutaneously for up to 2h post-injection (during this time animals were conscious and allowed to move freely). Finally, an exponential function was fitted to the data and the GFR estimated based on the T½ of the FITC-sinistrin clearance.

**Renal histology**

Kidneys were paraffin-embedded, cut and stained using Sirius red staining (Sigma‒Aldrich) and Hematoxylin and eosin (H&E, Sigma‒Aldrich) staining. Pictures of 10X of non-overlapping fields were taken with an Olympus microscope (BX63; Olympus FRANCE Rungis, France). Sirius red staining and image J software (<https://imagej.net/ij/)> were used to quantify the area corresponding to collagen fibrils (*i.e.*, interstitial fibrosis) for each image. We imaged the whole kidney over three entire kidney sections for each mouse, with 10 to 20 non-overlapping images with a magnification of 10X. Sham mice had mostly 18 images (therefore 6 per kidney section) while CKD mice had 10 images (3 to 4 per kidney section) because of the kidney size. Perivascular images were systematically excluded from the analysis. To evaluate glomerular injury, images with a magnification of 400X were used. At least 30 H&E-stained glomerular hilar cross-sections were analyzed from each kidney sample to determine glomerular area and volume using the ImageJ software (https://imagej.net/ij/). Briefly, after identifying glomeruli with both arterioles and proximal tubule in the same cross section, the outline of the glomerular area was manually drawn allowing the measurement. The glomerular volume (GV) was calculated as GV =(β/k)(Am)3/2, where β=1.38 (shape coefficient for spheres), k=1.1 (size distribution coefficient), and Am the surface area of the glomerulus. Glomerulosclerosis assessment was performed on H&E-stained sections using Artificial Intelligence -assisted image analysis. Each glomerulus was given a glomerulosclerosis-score on a 5-point scale according to the amount of capillary tuft involvement: score 0 (normal), score 1 (>0 to <25% involvement), score 2 (≥25 to <50% involvement), score 3 (≥50 to <75% involvement), and score 4 (global, ≥75% involvement). A glomerulosclerosis index (GSI) was calculated per animal as the mean score for all glomeruli in one transverse section at the level of the pelvis.

**Immunofluorescence (IF) staining**

Kidney mouse tissue staining, paraffin embedding tissue was used. Type 1 Collagen (Col-1) formalin-fixed sections (5μm) were deparaffinized and antigen retrieval performed by boiling sections for 20 min in 10 mM sodium citrate buffer (pH 6.0). Sections were then incubated with 10% normal horse serum followed by overnight incubation with primary antibodies rabbit anti-Col-1 (Abcam, Cambridge, UK: Anti-Collagen I antibody Cat. [ab254113]). The secondary antibodies were diluted with 1:200 (Goat anti-Rabbit IgG (H+L) Cross-Adsorbed Secondary Antibody, Alexa Fluor™ 568; Catalog # A-11011, Thermo Fisher Scientific).

**Immunohistochemistry using single chromogen**

Immunohistochemistry (IHC) was performed using standard procedures. Kidney rats tissue staining, paraffin embedding tissue was used. Briefly, after antigen retrieval and blocking of endogenous peroxidase activity, slides were incubated with the primary antibody. The primary antibody was detected using a polymeric Horseradish Peroxidase-linker antibody conjugate. Next, the primary antibody was visualized using 3,3'-diaminobenzidine as chromogen. Finally, sections were counterstained in hematoxylin and cover slipped. Slides were scanned under a 20X objective in a ScanScope AT slide scanner (Aperio, Leica, Nanterre, France). The primary antibodies used were: Goat anti-type I Collagen (Southern Biotech, Cat. 1310-01, Birmingham, AL, US); Rabbit anti-CD68 [E3O7V] (Cell signaling Technology, Cat. 97778, Danvers, MA, US); Goat anti-Kim-1 (R&D systems, Cat. AF1817, Minneapolis, MN, US). IHC-positive staining was quantified by image analysis using the VIS software (Visiopharm, Hørsholm, Denmark). VIS protocols were designed to analyze the virtual slides in two steps: first a crude detection of tissue at low magnification (1X objective) to exclude the kidney capsule, and then detection of IHC-positive staining. The quantitative estimates of IHC-positive staining were calculated as an area fraction (AF).

**Measurement of uremic toxins**

Total concentrations of uremic toxins were quantified by ultra-high performance liquid chromatography with ultraviolet and fluorescence detection (UPLC-UV/FLD) as previously described.^24^

**Gene expression analysis**

Total RNAs from intestine were extracted using TRI Reagent (Sigma Aldrich). Purity and concentration of RNA were determined using NanodropOne (Ozyme) and quality checked using Bioanalyser (Agilent). First-strand cDNAs were synthesized from 1 µg of total RNAs using PrimeScript RT kit reagent kit (Perfect Real Time, Takara Bio Europe). Quantitative real-time PCR (qPCR) assays were performed on 1/20 diluted cDNA using TB Green Premix Ex Taq (Tli RNaseH plus, Takara Bio Europe) with Rotor-Gene 6000 (Qiagen) using TB Green Premix Ex Taq (Tli RNaseH plus, Takara Bio Europe) as previously described.^S16^ TATA-box binding protein (*Tbp*) was used as a reference gene to normalize the results. Results are the ratio of target mRNA levels to *Tbp* mRNA levels and are expressed as percentage of the Sham group values. Primers sequences are listed in Supplementary Table S14.

**Cecal metagenomics**

**DNA extraction**

DNA were extracted from 0.1g aliquots of the fecal samples from cecal using the 96-soil kit (Macherey-Nagel, Allentown, PA, US). Bead beating was done horizontally on a Vortex-Genie 2 (Sigma-Aldrich) at 2700rpm for 5 minutes. A minimum of one negative control was included per batch of samples from the DNA extraction and throughout the laboratory process (including sequencing).^S17^

**DNA sequencing**

Before library preparation, the DNA was quantified using the Quant-iT dsDNA BR assay (Invitrogen, Waltham, MA, US). The genomic DNA was normalized to around 30 ng/μl and 10 μl DNA were used for the library construction. The genomic DNA was enzymatically fragmented, and libraries were prepared using the Celero DNA-Seq Core Module Kit (Tecan; Männedor, Switzerland) on a Tecan DreamPrep NGS platform (Tecan). DNA fragments were amplified using 7 PCR cycles. Short and large DNA fragments were removed using double-sided magnetic bead size selection (AMPure XP, Beckman Coulter, Brea, CA, US). The final concentration for each library was quantified (NuQuant NGS Library Quantification; Tecan) and the fragment length distribution was evaluated using the Fragment Analyzer 5200 system (Agilent, Santa Clara, CA, US). The fragmented DNA was used for library construction using NEBNext Ultra Library Prep Kit for Illumina (New England Biolabs). The prepared DNA libraries were evaluated using Qubit 2.0 fluorometer quantitation and Agilent 2100 Bioanalyzer for the fragment size distribution. qPCR was used to determine the concentration of the final library before sequencing. The library was sequenced using 2x150 bp paired-end sequencing on an Illumina platform (New England Biolabs).

**Gene catalog and MGS definitions**

As a reference gene catalog, we used the Clinical Microbiomics (<https://clinical-microbiomics.com>) mouse fecal and cecum microbiome gene catalog (7 099 388 genes). For metagenomics species (MGS) abundance profiling, we used the Clinical Microbiomics MmMGSIII v.2.0 set of 878 MGS, which has highly coherent abundance and base compositions.^S18,S19^

To taxonomically annotate an MGS, we aligned its genes against the representative genome assemblies of Genome Taxonomy Database (GTDB)^S20^ using Basic Local Alignment Search Tool (BLAST)+ v. 2.12.0 and used rank-specific annotation criteria.^S2^ Specifically, we assigned a taxon to an MGS if at least M% of its genes were mapped to the taxon, and no more than D% of its genes were mapped to a different taxon. We only considered BLAST hits with an alignment length ≥ 100 bp ≥ 50% query coverage, and % identity ≥ Percent Identity (PID). For the present study we defined PID = (95, 95, 85, 75, 65, 55, 50, 45); M = (75, 75, 60, 50, 40, 30, 25, 20); and D= (10, 10, 10, 20, 20, 20, 20, 15) for subspecies, species, genus, family order, class, phylum and superkingdom, respectively. Finally, we processed each MGS with CheckM (v1.1.11)^S21^ and updated our annotation with the CheckM result if this resulted in a lower taxonomic rank.

**Sequencing data preprocessing**

Raw Fast Quality control (FASTQ) files were filtered to remove host contamination by discarding read pairs in which either read mapped to the human reference genome GRCh38 with Bowtie2 (v. 2.4.2).^S22^ Reads were then trimmed to remove adapters and bases with a Phred score below 20 using AdapterRemoval (v. 2.3.1).^S23^ Read pairs in which both reads passed filtering with a length of at least 100 bp were retained; these were classified as high-quality non-host (HQNH) reads.

**Mapping reads to the gene catalog**

HQNH reads were mapped to the gene catalog usin29g Burrows-Wheeler Aligner (BWA-MEM) (v. 0.7.17).^S24^ An individual read was considered uniquely mapped to a gene if the mapping quality (MAPQ) was ≥ 20 and the read aligned with ≥ 95% identity over ≥100 bp. However, if > 10 bases of the read did not align to the gene or extended beyond the gene, the read was considered unmapped. Reads meeting the alignment length and identity criteria but not the minimum MAPQ threshold were considered multi-mapped. Each read pair was counted as either uniquely mapped to a specific gene, if one or both individual reads were uniquely mapped to a gene, or multi-mapped, if neither read was uniquely mapped, and at least one was multi-mapped, or unmapped, if both individual reads were unmapped. If the two reads were each uniquely mapped to a different gene, the gene mapped by read 1 was counted but not the gene mapped by read 2. A gene count table was created with the number of uniquely mapped read pairs for each gene.

**MGS relative abundance calculation**

For each MGS, a signature gene set was defined as the 100 genes optimized for accurate abundance profiling of the MGS. An MGS count table was created by counting the number of

reads uniquely mapped to the MGS signature genes per sample. An MGS was considered detected if reads from a sample uniquely mapped to at least three of its signature genes; measurements that did not satisfy this criterion were set to zero. Based on internal benchmarks, this threshold results in 99.6 % specificity. The MGS count table was normalized according to effective gene length and then normalized sample-wise to sum to 100%, resulting in relative abundance estimates for each MGS. Downsampled (rarefied) MGS abundance profiles were calculated by random sampling, without replacement, of a fixed number of signature gene counts per sample, and then following the procedure described above. In this study, 77 395 signature gene counts were sampled.

**Functional annotation and profiling**

EggNOG-mapper (v. 2.1.7; Diamond mode)^S25^ was used to map each gene in the gene catalog to the EggNOG orthologous group database (v. 5.0)^S26^, resulting in EggNOG annotations for 73% of genes and Kyoto Encyclopedia of Genes and Genomes (KEGG) orthology (KO) database annotations for 36% of genes. Functional potential profiles based on Kos were calculated as the proportion of all mapped reads that mapped to a given KO. KEGG modules (v. 78.2)^S27^ are defined as a set of Kos that enable a specific function or pathway. For each KEGG module, we defined its corresponding. Functional Species Group (FSG) as the set of MGSs that included at least 2/3 of the genes encoding the proteins/enzymes needed to complete the functionality of the module. If a module had alternative reaction paths, only one of these was required to be 2/3 complete. For modules with three or fewer steps, all steps were required to be comprised in the MGS. The Gut Metabolic Modules (GMMs) are a set of 103 conserved metabolic pathways, each defined as a series of enzymatic steps represented by KO identifiers.^S28^ We considered that an MGS contains a given module if the MGS included genes annotated to at least 2/3 of the KOs needed to complete the functionality of the module. If a module had alternative reaction paths, only one of these was required to be 2/3 complete. For modules with three or fewer steps, all steps were required to be comprised in the MGS. In general, three groups of genes associated with bile acid metabolism were defined, namely bile salt hydrolase (*bsh)*, the bai operon genes (*baiCDH*, *baiEI*, *baiN*), 7-alpha/beta-HSDH (*hsdh*) genes. To identify *bsh*, *bai*, and *hsdh* genes in the Clinical Microbiomics Mouse gene catalog (MmIII), reference protein sequences were gathered for 211 *bsh* genes from the 141 species^S29,S30^ for 68 bai operon genes, 31 *7-alpha-HSDH* genes and 5 *7-beta-HSDH* (described in the KEGG database https://www.genome.jp/pathway/map00121 and by Heinken *et al.*^S30^). Reference protein sequences were aligned to all proteins encoded in the Clinical Microbiomics Mouse gene catalog using BLAST v. 2.8.1 with 60% similarity and 80% coverage of the reference protein thresholds.^S2^ The similarity cut-off was empirically decided by observing that reference protein sequences and gene catalog protein sequences with identical Pfam annotations generally exhibited ≥60% similarity. Furthermore, the assignment catalog of genes to any of the reference proteins was confirmed by comparing functional domain profiles using Pfam v. 33.1. In total, 308, 26 and 91 genes were identified to represent *bsh*, bai operon genes (*baiCDH*, *baiEI, baiN*), and 7*-alpha-HSDH*, respectively.

**Diversity estimates**

Alpha and beta diversity estimates were calculated from rarefied abundance matrices, created by random sampling of reads without replacement. Within each data type (e.g., gene, MGS), all samples were represented by the same number of informative sequencing reads: rarefaction of MGS abundance was performed by sampling only from reads mapping to MGS signature genes, and rarefaction of KO abundance was performed by sampling only from reads mapped to a gene with an assigned KO. However, rarefaction of gene abundance was performed by sampling reads mapped to the entire gene catalog. Alpha diversity was calculated as the number of entities detected (richness), or as the Shannon index based on the natural logarithm. Beta diversity was calculated as the Bray-Curtis dissimilarity and used for Principal Coordinate Analysis (PCoA).

**Statistical Analysis**

Statistical analyses were performed in the R environment (<https://www.r-project.org>) and GraphPad Prism 10 (<https://www.graphpad.com>).

For animal tests, sample size was chosen on the basis of our earlier experience and no statistical test was used to predetermine sample size. All quantitative data are expressed as the mean ± standard error of the mean. Multiple comparisons were performed using one-way Analysis of Variance (ANOVA) followed, when appropriate, with Bonferroni *post hoc* tests for 2-by-2 comparisons. Results of the kinetic of generation of metabolites by SHIME were compared by two-way ANOVA (time, treatment). Simple comparisons were performed using a two-tailed Student’s t test.

For the analysis of the gut microbiota form the in vitro study, the linear discriminant analysis effect size (LEfSe), R package was used to identify the differentially abundant taxa and gut function between group. Features were detected with LEfSe if the following criteria were met: P ≤ 0.05 for Kruskal-Wallis and Wilcoxon tests and have an Linear Discriminant Analysis (LDA) score ≥ 2.0 or ≤ -2.0 .

For beta diversity, permutational multivariate analysis of variance (PERMANOVA) tests were performed using the adonis2 function from the vegan R package with 1000 permutations and by = “margin”, thus assessing the marginal effects of the terms (i.e. each marginal term analyzed in a model with all other variables). Multivariate homogeneity of group dispersion was tested using the betadisper and permutest functions from the vegan R package with 1000 permutations and type = “spatial median”, which is an implementation of the PERMDISP2 procedure.^S31^ With 1000 permutations, the lowest possible P value is 1/1001 = 0.000999.

For the analysis of gut microbiota from mice, down sampled microbiome functional profile, and taxonomic composition data were assessed between groups using non-parametric tests (Wilcoxon Mann-Whitney U test) corrected for multiple testing using the Benjamini-Hochberg false discovery rate (FDR) approach. FDR‐corrected q values < 0.1 were considered significantly different. Non-parametric directional standardized effect sizes were likewise taken as the Cliff’s delta and Spearman rho, respectively. Heatmap was used to compare the abundances (expressed by z‐scores) of all taxa between different groups. Results were considered significant when *P* <0.05.

**Supplementary References**

S1. Duranton F, Cohen G, De Smet R, et al. Normal and pathologic concentrations of uremic toxins. *J Am Soc Nephrol*. 2012;23(7):1258-1270. doi:10.1681/ASN.2011121175

S2. Altschul SF, Lipman DJ. Protein database searches for multiple alignments. *Proc Natl Acad Sci U S A*. 1990;87(14):5509-5513. doi:10.1073/pnas.87.14.5509

S3. Quevillon E, Silventoinen V, Pillai S, et al. InterProScan: protein domains identifier. *Nucleic Acids Res*. 2005;33(Web Server issue):W116-120. doi:10.1093/nar/gki442

S4. De MAN JC, Rogosa M, Sharpe ME. A Medium for the Cultivation of Lactobacilli. *Journal of Applied Bacteriology*. 1960;23(1):130-135. doi:10.1111/j.1365-2672.1960.tb00188.x

S5. Duboux S, Pruvost S, Joyce C, et al. The Pleiotropic Effects of Carbohydrate-Mediated Growth Rate Modifications in Bifidobacterium longum NCC 2705. *Microorganisms*. 2023;11(3):588. doi:10.3390/microorganisms11030588

S6. Hoefman S, Pommerening-Röser A, Samyn E, De Vos P, Heylen K. Efficient cryopreservation protocol enables accessibility of a broad range of ammonia-oxidizing bacteria for the scientific community. *Res Microbiol*. 2013;164(4):288-292. doi:10.1016/j.resmic.2013.01.007

S7. Molly K, Vande Woestyne M, Verstraete W. Development of a 5-step multi-chamber reactor as a simulation of the human intestinal microbial ecosystem. *Appl Microbiol Biotechnol*. 1993;39(2):254-258. doi:10.1007/BF00228615

S8. Van den Abbeele P, Grootaert C, Marzorati M, et al. Microbial community development in a dynamic gut model is reproducible, colon region specific, and selective for Bacteroidetes and Clostridium cluster IX. *Appl Environ Microbiol*. 2010;76(15):5237-5246. doi:10.1128/AEM.00759-10

S9. Possemiers S, Verthé K, Uyttendaele S, Verstraete W. PCR-DGGE-based quantification of stability of the microbial community in a simulator of the human intestinal microbial ecosystem. *FEMS Microbiol Ecol*. 2004;49(3):495-507. doi:10.1016/j.femsec.2004.05.002

S10. De Weirdt R, Possemiers S, Vermeulen G, et al. Human faecal microbiota display variable patterns of glycerol metabolism. *FEMS Microbiol Ecol*. 2010;74(3):601-611. doi:10.1111/j.1574-6941.2010.00974.x

S11. de Wiele TV, Boon N, Possemiers S, Jacobs H, Verstraete W. Prebiotic effects of chicory inulin in the simulator of the human intestinal microbial ecosystem. *FEMS Microbiol Ecol*. 2004;51(1):143-153. doi:10.1016/j.femsec.2004.07.014

S12. Vanden Bussche J, Marzorati M, Laukens D, Vanhaecke L. Validated High Resolution Mass Spectrometry-Based Approach for Metabolomic Fingerprinting of the Human Gut Phenotype. *Anal Chem*. 2015;87(21):10927-10934. doi:10.1021/acs.analchem.5b02688

S13. De Paepe E, Van Meulebroek L, Rombouts C, et al. A validated multi-matrix platform for metabolomic fingerprinting of human urine, feces and plasma using ultra-high performance liquid-chromatography coupled to hybrid orbitrap high-resolution mass spectrometry. *Anal Chim Acta*. 2018;1033:108-118. doi:10.1016/j.aca.2018.06.065

S14. Vandeputte D, Kathagen G, D’hoe K, et al. Quantitative microbiome profiling links gut community variation to microbial load. *Nature*. 2017;551(7681):507-511. doi:10.1038/nature24460

S15. Koppe L, Nyam E, Vivot K, et al. Urea impairs β cell glycolysis and insulin secretion in chronic kidney disease. *J Clin Invest*. 2016;126(9):3598-3612. doi:10.1172/JCI86181

S16. Barba C, Benoit B, Bres E, et al. A low aromatic amino-acid diet improves renal function and prevent kidney fibrosis in mice with chronic kidney disease. *Sci Rep*. 2021;11(1):19184. doi:10.1038/s41598-021-98718-x

S17. Qin J, Li Y, Cai Z, et al. A metagenome-wide association study of gut microbiota in type 2 diabetes. *Nature*. 2012;490(7418):55-60. doi:10.1038/nature11450

S18. Madsen MSA, Holm JB, Pallejà A, et al. Metabolic and gut microbiome changes following GLP-1 or dual GLP-1/GLP-2 receptor agonist treatment in diet-induced obese mice. *Sci Rep*. 2019;9(1):15582. doi:10.1038/s41598-019-52103-x

S19. Nielsen HB, Almeida M, Juncker AS, et al. Identification and assembly of genomes and genetic elements in complex metagenomic samples without using reference genomes. *Nat Biotechnol*. 2014;32(8):822-828. doi:10.1038/nbt.2939

S20. Parks DH, Chuvochina M, Rinke C, Mussig AJ, Chaumeil PA, Hugenholtz P. GTDB: an ongoing census of bacterial and archaeal diversity through a phylogenetically consistent, rank normalized and complete genome-based taxonomy. *Nucleic Acids Res*. 2022;50(D1):D785-D794. doi:10.1093/nar/gkab776

S21. Parks DH, Imelfort M, Skennerton CT, Hugenholtz P, Tyson GW. CheckM: assessing the quality of microbial genomes recovered from isolates, single cells, and metagenomes. *Genome Res*. 2015;25(7):1043-1055. doi:10.1101/gr.186072.114

S22. Langmead B, Salzberg SL. Fast gapped-read alignment with Bowtie 2. *Nat Methods*. 2012;9(4):357-359. doi:10.1038/nmeth.1923

S23. Schubert M, Lindgreen S, Orlando L. AdapterRemoval v2: rapid adapter trimming, identification, and read merging. *BMC Res Notes*. 2016;9:88. doi:10.1186/s13104-016-1900-2

S24. Li H, Durbin R. Fast and accurate short read alignment with Burrows-Wheeler transform. *Bioinformatics*. 2009;25(14):1754-1760. doi:10.1093/bioinformatics/btp324

S25. Cantalapiedra CP, Hernández-Plaza A, Letunic I, Bork P, Huerta-Cepas J. eggNOG-mapper v2: Functional Annotation, Orthology Assignments, and Domain Prediction at the Metagenomic Scale. *Mol Biol Evol*. 2021;38(12):5825-5829. doi:10.1093/molbev/msab293

S26. Huerta-Cepas J, Szklarczyk D, Heller D, et al. eggNOG 5.0: a hierarchical, functionally and phylogenetically annotated orthology resource based on 5090 organisms and 2502 viruses. *Nucleic Acids Res*. 2019;47(D1):D309-D314. doi:10.1093/nar/gky1085

S27. Kanehisa M, Goto S. KEGG: kyoto encyclopedia of genes and genomes. *Nucleic Acids Res*. 2000;28(1):27-30. doi:10.1093/nar/28.1.27

S28. Vieira-Silva S, Falony G, Darzi Y, et al. Species-function relationships shape ecological properties of the human gut microbiome. *Nat Microbiol*. 2016;1(8):16088. doi:10.1038/nmicrobiol.2016.88

S29. Song Z, Cai Y, Lao X, et al. Taxonomic profiling and populational patterns of bacterial bile salt hydrolase (BSH) genes based on worldwide human gut microbiome. *Microbiome*. 2019;7(1):9. doi:10.1186/s40168-019-0628-3

S30. Heinken A, Ravcheev DA, Baldini F, Heirendt L, Fleming RMT, Thiele I. Systematic assessment of secondary bile acid metabolism in gut microbes reveals distinct metabolic capabilities in inflammatory bowel disease. *Microbiome*. 2019;7(1):75. doi:10.1186/s40168-019-0689-3

S31. Anderson MJ, Ellingsen KE, McArdle BH. Multivariate dispersion as a measure of beta diversity. *Ecol Lett*. 2006;9(6):683-693. doi:10.1111/j.1461-0248.2006.00926.x
